# Beyond Tobacco Prevention: The Effects of Tobacco 21 Laws on Young Adults' Body Weight

**DOI:** 10.1002/hec.70120

**Published:** 2026-06-22

**Authors:** Qihua Qiu, Jaesang Sung

**Affiliations:** ^1^ James M. Hull College of Business Augusta University Augusta Georgia USA; ^2^ Department of Economics, Finance, and Accounting W. A. Franke College of Business Northern Arizona University Flagstaff Arizona USA

**Keywords:** body mass index, body weight, obesity, overweight, tobacco 21

## Abstract

Tobacco 21 (T21) laws effectively reduce youth tobacco use by preventing initiation. This study examines their impact on body weight among young adults aged 18–20. Using 2009–2019 Behavioral Risk Factor Surveillance System data and a two‐way fixed‐effects difference‐in‐differences (DID) design, we find limited evidence of broad weight changes in either direction across the BMI distribution. Obesity declines due to modest weight reductions concentrated near the upper BMI threshold, with no significant changes in overweight status or average BMI. Event study shows that the obesity decline emerges in the first post‐T21 year and attenuates afterward. Results are robust to alternative specifications, including an imputation DID approach addressing staggered adoption. Effects are driven by “never smokers”, consistent with a prevention‐based pathway, and are more pronounced among males and non‐White individuals, with heterogeneity observed across education levels in the upper BMI tail. Supplemental analyses using Youth Risk Behavior Survey data show reduced adverse weight outcomes among high schoolers aged 18+. T21 laws increase exercise, improve diets, and reduce sedentary behavior, underage drinking, marijuana use, and mental distress. Overall, T21 laws avoid the typical cessation‐related weight gain and modestly improve weight outcomes among at‐risk young adults, suggesting broader public health benefits beyond tobacco prevention.

## 1 Introduction

Obesity, defined as a Body Mass Index (BMI) ≥ 30,^1^ has become a major public health concern in the United States (C. Courtemanche 2009). Adult obesity rates rose from 13.4% in the 1960s to 42.8% in the late 2010s, and the prevalence of “overweight or obese” (BMI ≥ 25) increased from 44.9% to 73.1% (Fryar et al. 2020). Adolescent obesity increased from 4.6% to 21.2% over the same period (Fryar et al. 2018). Obesity elevates risks of chronic diseases (Cawley 2015) and has been linked to an estimated 300,000 premature deaths annually in the early 2000s (Flegal et al. 2004), second only to tobacco (Mokdad et al. 2004), and this number continues to grow (Global Burden of Disease Collaborative Network 2024). The associated economic burden is substantial, with direct medical costs estimated at $261 billion and total costs reaching $422 billion annually (Cawley et al. 2021; Woods and Miljkovic 2022).

Tobacco use remains the leading cause of preventable death in the U.S., with cigarette smoking alone accounting for over 480,000 deaths annually (U.S. Department of Health and Human Services 2014). Broad tobacco regulations, such as excise taxes, smokefree laws, and flavor bans, have contributed to substantial declines in tobacco use over recent decades (Evans et al. 1999; Tauras 2006; Carpenter and Cook 2008; DeCicca and McLeod 2008; Carton et al. 2016; C. J. Courtemanche et al. 2017; C. D. Cotti, Courtemanche, et al. 2024).^2^ Adult smoking prevalence fell from 42.4% in 1965 to 11.5% in 2021 (Centers for Disease Control and Prevention CDC 1999; Cornelius et al. 2023). Yet 18.7% of adults and 10% of middle and high school students still report current tobacco use (Cornelius et al. 2023; Birdsey et al. 2023).

Nearly 90% of daily smokers initiate before age 18, and 99% before age 26 (U.S. Department of Health and Human Services 2014), making tobacco prevention among youth and young adults critical for reducing long‐run tobacco use (Friedson et al. 2024). Broad tobacco regulations covering all age groups have been shown to reduce youth smoking (Carpenter and Cook 2008; DeCicca et al. 2008; C. J. Courtemanche et al. 2017; Titus et al. 2021). Among others, Tobacco 21 (T21) laws, which raise the minimum legal purchasing age (MLPA) for tobacco products to 21,^3^ directly target young adults aged 18–20 and have proven highly effective (Friedman and Wu 2020; Ali et al. 2020; Hansen et al. 2023; C. Cotti et al. 2024; Abouk et al. 2024; Friedman and Pesko 2024). By preventing early initiation, T21 laws are expected to reduce long‐run tobacco use and its associated health and economic costs.^4^

A related policy question is whether tobacco regulations affect body weight. An established literature, largely focused on cigarette taxes and broad adult populations, documents mixed evidence. Some studies find weight loss from higher cigarette prices/taxes (Gruber and Frakes 2006; C. Courtemanche 2009; Wehby and Courtemanche 2012), while others report weight gain effects (Chou et al. 2004; Baum 2009; Tchernis et al. 2022). Worksite smoking bans and randomized cessation programs have also been linked to weight gain (Liu et al. 2010; C. Courtemanche et al. 2018). While this debate raises concerns that cessation‐oriented tobacco regulations may unintentionally increase adult body weight and offset some public health gains, much less is known about whether such concerns apply to prevention‐oriented, youth‐targeting tobacco regulations such as T21 laws.

Using 2009‐2019 Behavioral Risk Factor Surveillance System (BRFSS) data, we estimate the effects of statewide T21 laws on body weight among young adults aged 18–20. Two‐way fixed effects (TWFE) difference‐in‐differences (DID) estimates show that statewide T21 adoption reduces the probability of being obese by 2.1 percentage points, a 17.9% decline relative to pre‐treatment obesity prevalence for this age group in treated states. Event study results indicate a 2.0‐percentage‐point decline in obesity in the first post‐T21 year, with effects attenuating thereafter. There're no significant effects on average BMI or on the probability of being overweight or obese. Quantile regression estimates validate that T21 laws do not shift the overall BMI distribution; instead, effects are concentrated in the upper tail near the obesity cutoff for this sample. These findings are robust to various robustness checks and falsification tests, including modern DID methods that address staggered policy adoption, such as imputation DID estimators (Borusyak et al. 2024).

We further document heterogeneity in T21's body weight effects, with more pronounced obesity reductions found among males and non‐White individuals. We observe a decrease in obesity among individuals with a high school diploma (HSD) and a decrease in “overweight or obese” among those without one. The effects are driven primarily by “never smokers”, consistent with evidence that T21 laws work mainly by preventing initiation rather than increasing cessation (Hansen et al. 2023; C. Cotti et al. 2024). This pattern points to a “prevention effect”, whereby avoiding early smoking initiation supports healthier lifestyle formation for weight management. A “spillover effect” may also play a role, as even those who would not have initiated smoking regardless may benefit from healthier peer environments (C. Courtemanche 2009).

Supplemental analyses using the 2009–2019 Youth Risk Behavior Surveys (YRBS) also find reductions in adverse weight outcomes among high school students aged 18+, while effects among those aged 15–17 are weaker and transitory. To explore mechanisms, we examine weight‐related behaviors using multiple data sources. BRFSS results show increases in exercise participation and fruit/vegetable consumption and reduced underage drinking and mental distress. YRBS results indicate declines in excessive TV watching, heavy soda consumption, and marijuana use. Evidence from the 2009‐2019 American Time Use Survey (ATUS) data further shows increased exercise time, reduced sedentary leisure, and more time eating at home rather than dining out, all consistent with improved weight outcomes.

As the first study to examine the impact of T21 laws on body weight, this paper extends the tobacco‐obesity literature by exploring a youth‐centered, prevention‐oriented tobacco regulation. Prior studies largely frame the tobacco‐obesity link through cessation‐driven mechanisms in broad adult populations, with youth and young adults typically appearing only as part of samples. While related work has examined youth physical activity (Choudhury and Conway 2020), body weight of young children with smoking‐age parents (Mellor 2011), and the effect of body weight on youth smoking initiation (Cawley et al. 2004; Rees and Sabia 2010), the direct effects of tobacco regulations on the body weight of smoking‐age youth and young adults remain understudied, especially for prevention‐oriented policies. By studying T21 laws, we highlight youth and young adults and show that such prevention‐oriented regulations do not induce adverse weight gain often linked to cessation. Rather, while average body weight is not significantly changed, T21 laws appear to promote healthier lifestyles among young adults at risk of obesity and reduce extreme adverse weight outcomes.

More broadly, our study speaks to a question that interests health economists beyond tobacco issues: whether early‐life, prevention‐oriented policies generate larger welfare gains than corrective interventions targeting already‐formed bad habits. From a policy perspective, our findings imply that youth‐focused substance prevention regulations might yield public health benefits beyond their primary goals and can be cost‐effective complements to traditional interventions.

## 2 Background

### 2.1 T21 Laws and Tobacco Use

The U.S. has regulated the MLPA for tobacco since the 19th century, with age limits varying by states and over time. After decades of advocacy, modern T21 laws began locally in Needham, Massachusetts, in 2005 (Reynolds et al. 2019). By 2015, over 100 localities had adopted T21 laws, including 79 in Massachusetts, 16 in New Jersey, and New York City. Hawaii became the first state to enact a statewide T21 law in January 2016, followed by California in June 2016, the District of Columbia and New Jersey in 2017, and Oregon, Maine, and Massachusetts in 2018. In 2019, 10 more states adopted statewide T21 laws before the federal law was enacted on December 20.^5^

Early local and statewide T21 laws (e.g., Hawaii, California) have been shown to reduce youth tobacco use and product sales (Friedman and Wu 2020; Ali et al. 2020; Glover‐Kudon et al. 2021; Grube et al. 2022; Wilhelm et al. 2022). Using 2009‐2019 BRFSS data, Hansen et al. (2023) provide the first comprehensive economic analysis of statewide T21 laws, finding significant reductions in smoking participation and daily smoking among young adults aged 18–20. Their YRBS analysis shows declines in frequent and daily smoking and e‐cigarette use among high school students aged 18+, along with spillover effects on younger adolescents and reductions in marijuana and alcohol use in some groups. They find no evidence of increased cessation, implying that the reduced smoking participation is driven by reduced initiation.

Other notable studies include C. Cotti et al. (2024), Abouk et al. (2024), and Friedman and Pesko (2024). Using longitudinal data from the Population Assessment of Tobacco Use and Health (PATH), C. Cotti et al. (2024) find that T21 laws reduce smoking among 18‐ to 20‐year‐olds. The panel structure of PATH allows them to show that these declines are mostly driven by fewer “never smokers” initiating than by increased cessation, a finding consistent with Hansen et al. (2023). Abouk et al. (2024) use Monitoring the Future (MTF) survey data and find that T21 laws reduce cigarette and e‐cigarette use among 12th graders. Their analysis of Nielsen Retail Scanner data shows reduced cigarette sales in counties with larger under‐21 populations. Friedman and Pesko (2024) show that greater T21 population coverage reduces tobacco use among 18‐ to 20‐year‐olds, especially when “possession, use, or purchase” (PUP) penalties are not enforced together.

More recent studies have examined T21's effects on maternal smoking. Using National Vital Statistics System (NVSS) data, Bersak et al. (2025) find modest but statistically significant reductions in smoking among mothers aged 18‐20 before and during pregnancy following T21 adoption, driven primarily by fewer women entering pregnancy as smokers rather than increased cessation during pregnancy. Also using the NVSS data but employing alternative identification strategies, Flynn (2025) finds no evidence that T21 laws reduce prenatal smoking or improve birth outcomes.

### 2.2 Smoking, Tobacco Regulations, and Body Weight

Identifying the causal effects of smoking on body weight is challenging due to confounding factors and potential reverse causality. Economists often address this by using exogenous instruments for smoking, most commonly tobacco regulations like cigarette taxes. However, findings remain mixed. Several U.S. studies find that higher cigarette taxes/prices reduce smoking but increase BMI and obesity (Chou et al. 2004; Nonnemaker et al. 2009; Baum 2009; Tchernis et al. 2022),^6^ while others, using same data sources (e.g., BRFSS, NLSY79) but alternative model specifications, find reductions in both smoking and BMI (Gruber and Frakes 2006; C. Courtemanche 2009; Wehby and Courtemanche 2012). C. J. Courtemanche et al. (2016) point out that the results appear sensitive to the set of economic controls included. Other regulations and interventions, such as worksite smoking bans and randomized cessation programs, generally reduce smoking but increase body weight (Liu et al. 2010; C. Courtemanche et al. 2018).

Findings on weight‐related behaviors are similarly mixed. Some studies find that cigarette taxes increase overall food consumption (Rozema and Ziebarth 2017), while others report improvements in diet quality such as increased fruit/vegetable intake (C. Courtemanche 2009; Wehby and Courtemanche 2012). Several studies suggest cigarette taxes reduce exercise (Conway and Niles 2017; Choudhury and Conway 2020), whereas others find the opposite (C. Courtemanche 2009; Wehby and Courtemanche 2012).

Despite mixed findings, prior studies typically link tobacco regulations to body weight by assuming their effects operate primarily through promoting cessation among established smokers. Several mechanisms have been proposed. On the negative side, quitting smoking can slow metabolism and increase food cravings from nicotine withdrawal (Williamson et al. 1991; Nicklas et al. 1999) and may lead to “oral fixation” whereby individuals substitute food for cigarettes (Loud et al. 2022). It may also trigger compensatory behaviors such as increased alcohol consumption (Koksal and Wohlgenant 2016) or reduced exercise (deRuiter and Faulkner 2006), often rationalized by “compensatory health beliefs”, the idea that quitting smoking justifies engaging in other unhealthy behaviors (Radtke et al. 2011, 2012).

On the positive side, quitting smoking may motivate healthier behaviors, such as more exercise and better diets, to avoid post‐cessation weight gain (C. Courtemanche 2009). Improved lung capacity may also make exercise more enjoyable (Hedenström et al. 1986). In addition, savings from reduced tobacco spending can facilitate healthier consumption choices of food and fitness (Busch et al. 2004). Successfully resisting smoking may further strengthen willpower, helping individuals resist other unhealthy temptations (Ozdenoren et al. 2012). Supporting this view, studies have found strong complementarity between smoking and alcohol consumption (Dee 1999; Picone and Sloan 2003).

While prior studies offer important insights for the general population, evidence for youth and young adults remains limited. Some research has examined how body weight affects youth smoking initiation (Cawley et al. 2004; Rees and Sabia 2010), yet few explore the reverse. Mellor (2011) analyzes how cigarettes taxes affect the body weight of young children with smoking‐age parents, yet direct evidence for smoking‐age youths and young adults themselves remains scarce. Therefore, understanding how early‐life, prevention‐oriented tobacco regulations, such as the T21 laws, affect body weight among this young population fills an important gap in the literature.

### 2.3 How T21 Laws Might Affect Body Weight

T21 laws work primarily by preventing initiation among youth and young adults, most of whom have never smoked and are still forming long‐term health habits. Therefore, the pathways linking broad tobacco regulations to body weight, typically through promoting cessation among established smokers, may not fully apply in this setting.

First, because T21 laws primarily work by preventing initiation rather than increasing cessation, metabolic disruptions and food cravings associated with nicotine withdrawal are largely avoided. While nicotine withdrawal is linked to weight gain (Nicklas et al. 1999), initiating nicotine use does not necessarily lead to weight loss. Recent medical evidence shows that smokers tend to accumulate more abdominal fat than non‐smokers, challenging the notion that smoking always promotes weight loss (Carrasquilla et al. 2024).

Second, by deterring smoking initiation, T21 laws may generate a “prevention effect” that helps young adults avoid adopting downstream unhealthy behaviors, such as overeating, underage drinking, and physical inactivity, that can undermine weight management. In other words, young adults without exposure to T21 laws are more likely to initiate smoking and subsequently engage in these risky behaviors, potentially leading to weight gain, compared to their T21‐treated peers. This aligns with previously discussed pathways where smoking can impair lung capacity, weaken willpower, diminish health prioritization, and strain financial resources, all of which can make it more difficult to maintain healthy habits.

T21 laws may also generate a “spillover effect” even among “true never smokers” who are unlikely to have initiated smoking regardless. People's eating and exercise behaviors are strongly influenced by those around them (Christakis and Fowler 2007), and peer influence is especially pronounced during young adulthood. A more health‐conscious peer environment, marked by less smoking and other downstream risky behaviors but better dietary and exercise habits, may therefore encourage young adults to keep healthy lifestyles. Similar spillovers have been documented; for instance, C. Courtemanche (2009) finds that cigarette taxes can reduce body weight among non‐smokers when their peers smoke less and adopt healthier habits.

Supporting these pathways, Hansen et al. (2023) show that T21 laws reduce alcohol and marijuana use among some young adults, indicating broader behavioral improvements beyond tobacco prevention. While not studying T21 laws, Conway and Niles (2017) find that cigarette taxes increase exercise among “never‐smoking” young adults, suggesting that even broad tobacco regulations may have a “prevention effect” in this age group.

## 3 Data

### 3.1 BRFSS

We use data from the 2009‐2019 Behavioral Risk Factor Surveillance System (BRFSS), a nationally representative cross‐sectional survey of U.S. adults that collects information on chronic health conditions, risky behaviors, and preventive care utilization. We focus on young adults aged 18–20, the age group directly subject to T21 laws.

The main outcomes include BMI (kg/m^2^) and two binary indicators of adverse weight status: “overweight or obese” (BMI ≥ 25) and “obese” (BMI ≥ 30). We exclude observations in the bottom and top 1% of the BMI distribution to remove outliers. We also examine weight‐related behaviors (all in binary form), including exercise participation, daily fruit and vegetable intake meeting USDA guidelines,^7^ current smoking, daily smoking, current drinking, binge drinking, and frequent mental distress (FMD), defined by the CDC as ≥14 mentally unhealthy days in the past 30 days. Supporting Information S1: Table A2, Column (1), reports weighted summary statistics for this BRFSS sample. The average BMI is 24.4; 35.7% of respondents are overweight or obese, and 12.4% are obese. About 85% report exercising in the past month, 29.4% meet USDA daily fruit intake guidelines, and 21% meet vegetable intake guidelines. Additionally, 11.8% are current smokers, 6.9% smoke daily,^8^ 33.6% are current drinkers, 15.5% report binge drinking, and 13.4% experience FMD.

Demographic covariates include binary indicators for age, gender, race/ethnicity, education, student status, and employment status, plus logarithmic group average income.^9^ In this sample, 16.6% do not have a high school diploma (HSD), 46.6% are high school graduates, and the rest have at least some college educations. Approximately 49% are students (not employed), and 37% are employed (not students).

### 3.2 YRBS

As a supplemental analysis, we follow Hansen et al. (2023) and use 2009–2019 waves of the State Youth Risk Behavior Surveys (YRBS), which collect information on risky behaviors among U.S. middle and high school students. The state YRBS is conducted biennially, mostly in odd‐numbered years. Our sample includes 44 states and 234 state‐years, as not all states participated in each wave. Among states with statewide T21 laws, Massachusetts, Ohio, Oregon, and the District of Columbia are not included in the state YRBS, leaving 13 “treated states” for analysis.

We focus on high school students aged 18+. Supporting Information S1: Table A2, Column (2), presents weighted summary statistics for this sample. The “overweight or obese” rate (35.8%) and average BMI (24.5) match those in the BRFSS sample, though the obesity rate is slightly higher (13.7%). These outcomes are based on standard adult BMI thresholds (BMI ≥ 25 for “overweight or obese”, BMI ≥ 30 for “obese”). The YRBS also provides age‐sex‐specific BMI percentiles and corresponding adverse weight indicators (≥ 85th percentile for “overweight or obese”, ≥ 95th for “obese”), which are commonly used for children and adolescents.

Regarding weight‐related behaviors, 81.1% of students report exercising at least one day per week,^10^ 29.5% meet USDA daily fruit intake guidelines, and 19.3% meet vegetable intake guidelines. Additionally, 27.2% watch TV for three or more hours per school day, and 5.6% drink soda more than three times daily. Nearly 17.8% report current smoking, with 6.5% smoking frequently (≥ 20 days/month); 42.9% drink alcohol; 25.8% report marijuana use, with 12% reporting frequent marijuana use (≥ 10 times/month) (D. Anderson et al. 2015; D. M. Anderson and Rees 2023). Compared to BRFSS, the YRBS sample includes more non‐Hispanic blacks and Hispanics. Most respondents are in 12th grade (91%).

### 3.3 Additional Control Variables

We control for state‐level covariates, including population coverage of local T21 laws^11^; other tobacco regulations: cigarette taxes, population coverage of comprehensive indoor smoking restriction (ISR) and indoor vaping restrictions (IVR) by venue (workplaces, restaurants, bars) (Seidenberg et al. 2024), presence of indoor smoking bans on campus (K‐12, university/college), standardized e‐cigarette taxes (C. Cotti, Nesson et al. 2024), and presence of e‐cigarette MLPA at 18; alcohol and marijuana regulations: beer excise taxes and presence of medical and recreational marijuana laws; weight‐related policies: gasoline taxes, soda and food sales taxes, and high school physical education requirements, nutrition education standards, and beverage nutrition standards by school venue (cafeterias, vending machines, school stores, fundraisers); and economic conditions: personal income per capita and unemployment rates.^12^ All monetary variables are adjusted to 2017 dollars using the Personal Consumption Expenditures Price Index.

## 4 Methods

For the BRFSS analysis, we estimate the following TWFE DID model:

(1) $$Y_{ist}=\beta_{0}+\beta_{1}T21_{st}+\beta_{2}X_{ist}+\beta_{3}Z_{st}+\delta_{s}+\tau_{t}+\varepsilon_{ist}$$

where $Y_{ist}$ denotes the outcome of interest for individual i in state s and year‐by‐month t. $T21_{st}$ is a binary indicator for the presence of a statewide T21 law; $\beta_{1}$ captures the average treatment effects on the treated (ATT). $X_{ist}$ are individual demographics (see Section 3.1) and age‐specific trends (Hansen et al. 2023). $Z_{st}$ are state‐level covariates (see Section 3.3). State fixed effects, $\delta_{s}$, absorb time‐invariant state‐level unobservable (e.g., cultural norms). Year‐by‐month fixed effects, $\tau_{t}$, capture common national shocks and seasonality. $\varepsilon_{ist}$ denotes idiosyncratic errors. All regressions apply BRFSS sampling weights and cluster robust standard errors at the state level.

For continuous outcomes like BMI, we estimate Equation (1) using ordinary least squares (OLS). For binary body weight outcomes, we use linear probability models (LPM) throughout for computational convenience.^13^ For weight‐related behavioral outcomes (all in binary form), some of which have very low prevalence in later years (e.g., daily smoking < 3% in 2019),^14^ so we report marginal effects from Logit models as our preferred specification and also display LPM estimates for comparison.

Body weight reflects cumulative calorie balance, so policy effects may evolve over time. To capture dynamic responses, we estimate an event study specification that replaces $T21_{st}$ with binary indicators for post‐T21 periods (the first year, and the second year onward). We also include pre‐event indicators for the second, the third, and the fourth year onward before T21 adoption, using the first year immediately prior as the reference period. These pre‐event estimates allow us to assess the parallel trends assumption, which posits that, absent T21 laws, trends in body weight would have been similar across states.^15^

For the supplemental YRBS analysis, we estimate Equation (1) with t denoting year rather than year‐by‐month. $T21_{st}$ is defined as the share of months in year t that a statewide T21 law was in effect (Hansen et al. 2023). We also report corresponding event study estimates.

## 5 Results

### 5.1 BRFSS Findings

#### 5.1.1 DID Analysis

**FIGURE 1:**
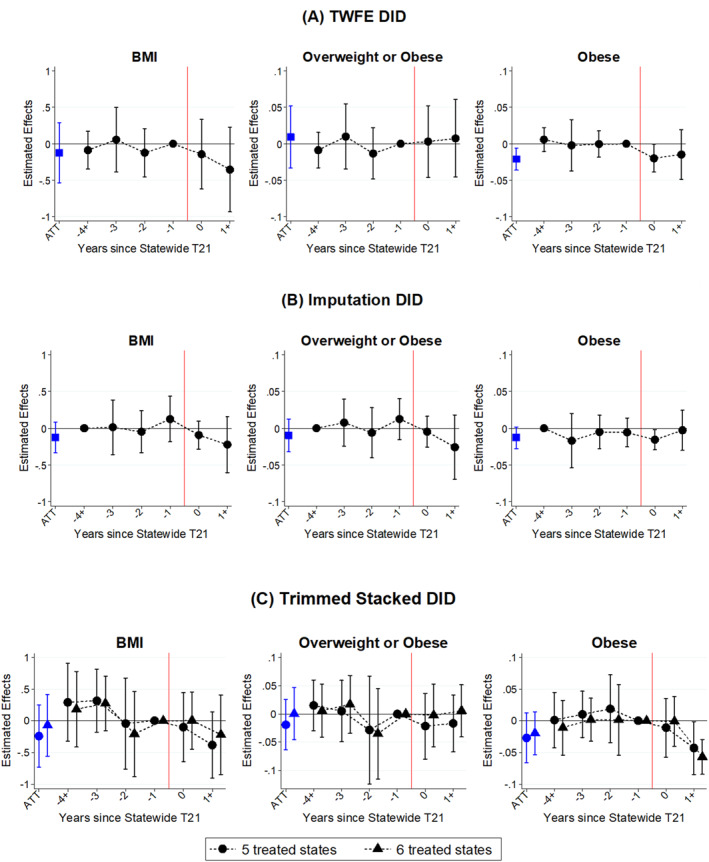
Effects of T21 laws on body weight among young adults aged 18–20, BRFSS, 2009–2019. ATT: average treatment effects on the treated; BMI: body mass index; DID: difference‐in‐differences; TWFE: two‐way fixed‐effects.

Body weight reflects long‐term calorie balance, so its response to the adoption of T21 laws may evolve gradually, making it important to examine dynamic effects first. Figure 1, Panel (A), presents event study estimates using the full set of controls from Equation (1), with solid circles indicating point estimates and bars representing 95% confidence intervals. We find no statistically significant post‐treatment effects on average BMI or on the probability of being overweight or obese. The probability of being obese declines by 2 percentage points in the first year following T21 adoption, with the effect attenuating and becoming statistically insignificant thereafter. The statistically insignificant pre‐event estimates support the parallel trends assumption, suggesting that the observed post‐T21 changes in obesity are not driven by pre‐existing differences between treated and control states.

Figure 1, Panel (A), also displays ATT estimates (solid squares and bars), showing a statistically significant reduction in obesity. Table 1 presents ATT estimates using a built‐up strategy that sequentially adds controls. Column (1) begins with a parsimonious TWFE specification including only state and year‐by‐month fixed effects. Column (2) adds individual demographics. Column (3) further incorporates local T21 population coverage. All estimates in Columns (1)–(3) are statistically insignificant. Column (4) adds other tobacco control regulations, tripling the estimated effect on obesity and making it statistically significant, which is expected given the strong correlation between a state's broader tobacco control environment and statewide T21 adoption. Columns (5)–(7) sequentially add controls for alcohol and marijuana regulations, other weight‐related policies, and economic conditions, with little change in the estimated effect on obesity. Interpreting the ATT estimate in Column (7), which is also plot in Figure 1, statewide T21 implementation reduces the probability of being obese among 18‐ to 20‐year‐olds by 2.1 percentage points, a 17.9% decline relative to pre‐treatment obesity prevalence among this age group in treated states. For robustness check, Column (8) adds state‐specific time trends to capture unobserved, time‐varying state factors, slightly attenuating but not eliminating the statistically significant effect on obesity.^16^ Across all columns, we find no significant effects on average BMI or on the probability of being overweight or obese.

**TABLE 1:** Effects of T21 laws on body weight among young adults aged 18–20, BRFSS, 2009–2019, DID estimates.

Dependent variables	(1)	(2)	(3)	(4)	(5)	(6)	(7)	(8)
BMI	−0.112	−0.091	−0.088	−0.141	−0.151	−0.158	−0.123	−0.0001
	(0.107)	(0.100)	(0.097)	(0.216)	(0.220)	(0.199)	(0.205)	(0.232)
	[−0.5%]	[−0.4%]	[−0.4%]	[−0.6%]	[−0.6%]	[−0.7%]	[−0.5%]	[0.0%]
Overweight or obese	−0.0004	0.002	0.002	0.012	0.009	0.006	0.009	0.024
	(0.014)	(0.013)	(0.013)	(0.021)	(0.022)	(0.021)	(0.021)	(0.027)
	[−0.1%]	[0.7%]	[0.6%]	[3.4%]	[2.6%]	[1.8%]	[2.7%]	[6.9%]
Obese	−0.008	−0.007	−0.007	−0.023**	−0.022***	−0.022***	−0.021***	−0.016*
	(0.005)	(0.006)	(0.006)	(0.009)	(0.009)	(0.008)	(0.008)	(0.009)
	[−6.4%]	[−5.9%]	[−6.1%]	[−19.2%]	[−18.6%]	[−18.6%]	[−17.9%]	[−13.9%]
Demographics		Y	Y	Y	Y	Y	Y	Y
Local T21 coverage			Y	Y	Y	Y	Y	Y
Other tobacco policies				Y	Y	Y	Y	Y
Alcohol & marijuana policies					Y	Y	Y	Y
Weight‐related policies						Y	Y	Y
Economic conditions							Y	Y
State‐specific trends								Y

*Note:* Regressions include individuals in 50 states and the District of Columbia (*N* = 88,534). All regressions use BRFSS‐provided sampling weights. Robust standard errors in parenthesis are clustered at the state level. Percent changes relative to pre‐treatment means for treated states are reported in brackets. Demographics include binary indicators for respondent's age (along with age‐specific time trends), gender, race/ethnicity, educational attainment, student status, and employment status, as well as logarithmic group average income. Other tobacco policies include cigarette taxes, state population coverages of comprehensive ISR by venue (bars, restaurants, and workplaces), state population coverages of comprehensive IVR by venue (bars, restaurants, and workplaces), standardized e‐cigarette taxes, and presence of MLPA for e‐cigarettes at 18. Alcohol and marijuana policies include beer taxes, medical marijuana laws, and recreational marijuana laws. Other weight‐related controls include gasoline taxes, soda sales taxes, food sales taxes, state PE time requirements, state nutrition education standards, and state school beverage nutrition standards by venue (cafeterias, vending machines, school stores, fundraisers). Economic conditions include personal income per capita and unemployment rates. All regressions control state fixed effects and time (year‐by‐month) fixed effects.

* *p* < 0.1.

** *p* < 0.05.

*** *p* < 0.01.

#### 5.1.2 Robustness Checks for Staggered Adoption

A growing body of methodological work cautions that TWFE DID estimators may be biased under staggered policy adoption with heterogeneous treatment effects (De Chaisemartin and d’Haultfoeuille 2020; Goodman‐Bacon 2021; Callaway and Sant’Anna 2021; Sun and Abraham 2021; Baker et al. 2022). To assess the robustness of our findings, we apply two modern DID approaches designed for staggered adoption settings: the imputation DID estimator of Borusyak et al. (2024) and the trimmed stacked DID estimator following Wing et al. (2024).

We first apply the imputation DID estimator, which imputes counterfactual outcomes for treated units using untreated observations and aggregates treatment effects across cohorts and event‐time (horizons). One merit of this approach is that it retains all treated states and does not require trimming late adopters, making it particularly well suited to our context, where most treated states adopted statewide T21 laws in 2019. Figure 1, Panel (B), presents the event study and ATT estimates. By design of this approach, the “4 year onward pre‐T21” period serves as the reference period. The imputation DID estimates closely track our TWFE DID estimates but are slightly smaller in magnitude. The ATT estimate indicates a 1.2‐percentage‐point reduction in the probability of being obese. The event study shows a significant 1.6‐percentage‐point decline in obesity in the first post‐T21 year, with effects attenuating thereafter. There is no evidence of differential pre‐treatment trends.

We next implement the trimmed stacked DID approach of Wing et al. (2024). Following their recommended practice, we aggregate individual‐level BRFSS data to state‐level using survey weights and construct event‐time stacks centered on statewide T21 effective date. Late adopters are trimmed to ensure balanced pre‐ and post‐treatment windows across stacks, each constituting a canonical two‐by‐two DID comparison between treated states and not‐yet‐treated or never‐treated control states. We use a 60‐month (5‐year) pre‐treatment period and consider two post‐treatment specifications: (1) retaining five treated states with a 24‐month post‐treatment window, and (2) retaining six treated states with an 18‐month post‐treatment window. Both allow us to average monthly estimates into “the first post‐T21 year” and “thereafter”. Using the trimmed stacked samples, we estimate Weighted Least Squares regressions, with weights based on sample share as recommended by Wing et al. (2024).

Figure 1, Panel (C), presents the trimmed stacked DID results, which are qualitatively consistent with our main findings by showing significant declines in obesity after T21 adoption, though the effects emerge after the first post‐T21 year rather than immediately. Perhaps because we lose many treated states in trimming, the ATT are less precisely estimated and do not reach conventional significance levels. We therefore view these stacked DID results as merely complementary to the imputation DID results.^17^

#### 5.1.3 Other Robustness Checks and Falsification Tests

**FIGURE 2:**
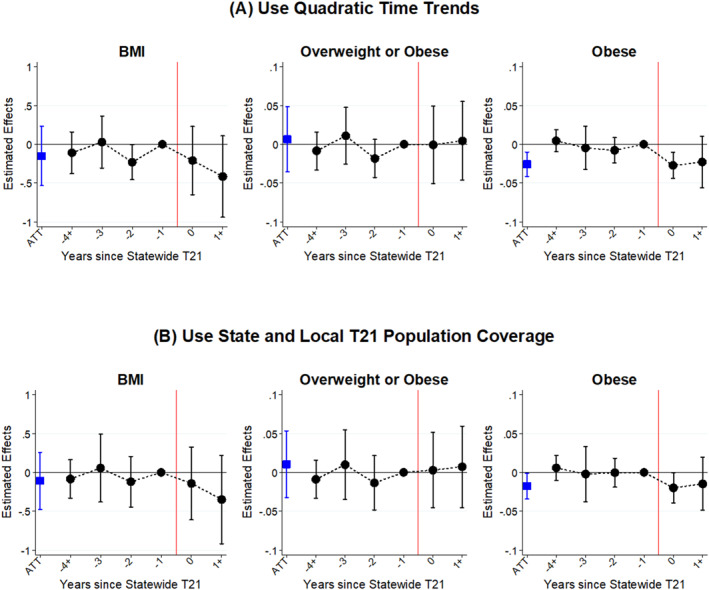
Effects of T21 laws on body weight among young adults aged 18–20, BRFSS, 2009–2019, additional robustness checks. ATT: average treatment effects on the treated; BMI: body mass index.

We conduct some additional robustness checks. First, we replace year‐by‐month fixed effects with quadratic year‐month trends. While prior studies show that such alternative time controls can flip the estimated effects of cigarette taxes/prices on body weight (Chou et al. 2004; Gruber and Frakes 2006), our results remain robust, as shown in Figure 2, Panel (A). Second, we alternatively define treatment using the share of the state's population covered by statewide or local T21 laws (Friedman and Pesko 2024). Figure 2, Panel (B), shows that our results still hold.^18^ Lastly, we conduct a “leave‐one‐treated‐state‐out” analysis. Supporting Information S1: Figure A3 displays ATT estimates for all body weight outcomes and event study estimates for obesity, showing that the results remain robust no matter which treated state is excluded, suggesting that no single state disproportionately drives our findings.

We perform two falsification tests. First, we pretend that statewide T21 adoptions occurred earlier in the sample period, such as 2013–2016 or 2012–2015, instead of the actual 2016–2019. As shown in Supporting Information S1: Figure A4, Panel (A), all placebo estimates are statistically insignificant. Second, we run a Fisher Randomization Test by randomly reassigning T21 adoption across states and re‐estimating Equation (1) 500 times. Supporting Information S1: Figure A4, Panel (B), plots the resulting placebo distributions of ATT estimates and t‐statistics, all centered near zero. The true ATT estimate and t‐statistic for “obese” (marked by red lines) lie in the right tail of these distributions, with only 1% of placebo ATT estimates and 0.6% of placebo t‐statistics exceeding the true values. This test provides strong non‐parametric evidence that our results are not driven by spurious trends in body weight but reflect the causal impact of T21 laws.

#### 5.1.4 Heterogeneous Effects

Our main results suggest that the effects of T21 laws among 18‐ to 20‐year‐olds are not uniform across the BMI distribution but are concentrated at the upper tail. To further characterize this heterogeneity, we estimate quantile regressions at BMI deciles (0.1, 0.2, …, 0.8, 0.9). As shown in Table 2, Panel A, T21 laws are associated with statistically significant BMI reductions of 0.58 and 1.12 units at the 0.8 and 0.9 quantiles, respectively, corresponding to relative declines of 2.1% and 3.6% from pre‐treatment BMI centile levels. These results indicate that T21 laws primarily affect individuals at higher risk of adverse weight outcomes, modestly reducing BMI near the obesity threshold without shifting the broader distribution.^19^

**TABLE 2:** Effects of T21 laws on BMI, 2009–2019, quantile regression estimates.

Quantile	0.1	0.2	0.3	0.4	0.5	0.6	0.7	0.8	0.9
Panel A: Age Group 18–20, BRFSS									
BMI	−0.235	0.039	0.236	0.240	0.105	0.361	0.070	−0.580**	−1.124**
	(0.204)	(0.150)	(0.159)	(0.164)	(0.158)	(0.228)	(0.272)	(0.295)	(0.525)
	[−1.2%]	[0.2%]	[1.1%]	[1.1%]	[0.5%]	[1.5%]	[0.3%]	[−2.1%]	[−3.6%]
Centile level	19.4	20.5	21.5	22.4	23.3	24.4	25.8	27.6	31.0
Panel B: Age Group 18+, YRBS									
BMI	−0.536	−0.232	−0.339	−0.468	−0.817*	−1.136**	−1.493**	−0.977	−1.092
	(0.329)	(0.341)	(0.389)	(0.379)	(0.436)	(0.507)	(0.596)	(0.731)	(1.005)
	[−2.8%]	[−1.1%]	[−1.6%]	[−2.1%]	[−3.5%]	[−4.7%]	[−5.8%]	[−3.5%]	[−3.5%]
Centile level	19.1	20.3	21.2	22.1	23.0	24.2	25.6	27.6	31.4
Panel C: Age Group 15–17, YRBS									
BMI	−0.067	−0.160	−0.206	−0.125	−0.192	−0.303*	−0.395**	−0.354	−0.992*
	(0.139)	(0.113)	(0.132)	(0.129)	(0.130)	(0.169)	(0.199)	(0.309)	(0.397)
	[−0.4%]	[−0.8%]	[−1.0%]	[−0.6%]	[−0.9%]	[−1.3%]	[−1.6%]	[−1.3%]	[−3.4%]
Centile level	18.5	19.6	20.4	21.2	22.1	23.0	24.3	26.2	29.6

*Note:* Regressions in Panel A include individuals in 50 states and the District of Columbia (*N* = 88,534). Regressions in Panel B include YRBS sampled high school students aged 18+ in 44 states (*N* = 86,472). Regressions in Panel C include YRBS sampled high school students aged 15–17 in 44 states (*N* = 662,144). All regressions use survey‐provided sampling weights. Robust standard errors in parenthesis are clustered at the state level. Percent changes relative to corresponding pre‐treatment centiles for treated states are reported in brackets. Regressions in Panel A control respondents' age (along with age‐specific time trends), gender, race/ethnicity, educational attainment, student status, and employment status, as well as logarithmic group average income. Regressions in Panels B and C control binary indicators for respondents' age and age‐specific time trends, gender, race/ethnicity, and grades. All regressions control cigarette taxes, state population coverages of comprehensive ISR by venue (bars, restaurants, and workplaces), state population coverages of comprehensive IVR by venue (bars, restaurants, and workplaces), standardized e‐cigarette taxes, presence of MLPA for e‐cigarettes at 18, beer taxes, medical marijuana laws, recreational marijuana laws, gasoline taxes, soda sales taxes, food sales taxes, state PE time requirements, state nutrition education standards, state school beverage nutrition standards by venue (cafeterias, vending machines, school stores, fundraisers), personal income per capita, and unemployment rates. Regressions in Panel A control state fixed effects and year‐by‐month fixed effects. Regressions in Panels B and C control state fixed effects and year fixed effects.

* *p* < 0.1.

** *p* < 0.05.

*** *p* < 0.01.

We next examine heterogeneity across demographic and socioeconomic subgroups. Table 3 reports subsample DID estimates by gender (male vs. female), race (Whites vs. non‐Whites), income (above vs. below the state median household income), and education attainment (with vs. without a high school diploma, HSD). Panel A and Panel B indicate that obesity reduction is more pronounced among males and non‐White young adults,^20^ respectively. Panel C shows statistically insignificant effects for both income groups. The larger point estimates for higher‐income individuals are accompanied by larger standard errors, thus yielding no statistical evidence of heterogeneity by income. Panel D shows a reduction in obesity among young adults with a HSD and a reduction in “overweight or obese” among those without one, indicating heterogenous effects at different points of the upper BMI distribution across education groups.

**TABLE 3:** Effects of T21 laws on body weight among young adults aged 18–20, BRFSS, 2009–2019, DID estimates, subsamples.

	BMI (1)	Overweight or obese (2)	Obese (3)	# Obs.
Panel A: Gender				
Male	−0.125 (0.190) [−0.5%]	−0.006 (0.023) [−1.5%]	−0.023 (0.010)** [−19.0%]	48,580
Female	−0.061 (0.313) [−0.3%]	0.036 (0.035) [11.7%]	−0.016 (0.018) [−14.2%]	39,954
Panel B: Race/Ethnicity				
White	−0.030 (0.309) [−0.1%]	0.013 (0.032) [4.1%]	−0.017 (0.012) [−15.5%]	56,514
Nonwhite	−0.185 (0.233) [−0.8%]	0.009 (0.038) [2.3%]	−0.025 (0.012)** [−19.5%]	32,020
Panel C: Income				
High income	−0.383 (0.553) [−1.6%]	−0.030 (0.053) [−9.7%]	−0.038 (0.025) [−38.0%]	23,730
Low income	−0.026 (0.203) [−0.1%]	0.018 (0.020) [5.0%]	−0.014 (0.010) [−11.6%]	64,804
Panel D: Education				
HSD or above	−0.117 (0.199) [−0.5%]	0.021 (0.021) [6.0%]	−0.023 (0.007)*** [−19.7%]	77,990
Less than HSD	−0.547 (0.510) [−2.3%]	−0.104 (0.053)* [−29.3%]	−0.026 (0.031) [−21.3%]	10,544
Panel E: Smoking status				
Never smokers	−0.073 (0.212) [−0.3%]	0.013 (0.024) [3.8%]	−0.021 (0.009)** [−17.9%]	71,663
Ever smokers	0.010 (0.475) [0.0%]	0.016 (0.051) [4.3%]	−0.015 (0.030) [−11.3%]	15,061

*Note:* Full sample includes individuals in 50 states and the District of Columbia (*N* = 88,534). All regressions use BRFSS‐provided sampling weights. Robust standard errors in parenthesis are clustered at the state level. Percent changes relative to pre‐treatment means for treated states are reported in brackets. Regressions control respondents' age (along with age‐specific time trends), gender, race/ethnicity, educational attainment, student status, employment status, logarithmic group average income, cigarette taxes, state population coverages of comprehensive ISR by venue (bars, restaurants, and workplaces), state population coverages of comprehensive IVR by venue (bars, restaurants, and workplaces), standardized e‐cigarette taxes, presence of MLPA for e‐cigarettes at 18, beer taxes, medical marijuana laws, recreational marijuana laws, gasoline taxes, soda sales taxes, food sales taxes, state PE time requirements, state nutrition education standards, state school beverage nutrition standards by venue (cafeterias, vending machines, school stores, fundraisers), personal income per capita, unemployment rates, state fixed effects, and year‐by‐month fixed effects.

^*^ *p* < 0.1.

^**^ *p* < 0.05.

^***^ *p* < 0.01.

Finally, motivated by evidence that T21 laws work primarily by preventing smoking initiation rather than increasing cessation (Hansen et al. 2023; C. Cotti et al. 2024), we examine whether their effects on body weight are also driven by “never smokers”. As shown in Table 3, Panel E, the reduction in obesity is expectedly concentrated among “never smokers”. While prior research finds limited effects of tobacco regulations on “never smokers” in broad adult samples (Nonnemaker et al. 2009), this need not apply to young adults who are still forming long‐term health habits. As discussed in Section 2.3, by deterring initiation among those who might otherwise have started smoking, T21 laws may generate a “prevention effect” that helps avert downstream consequences, such as impaired lung capacity, weakened willpower, diminished health prioritization, and financial strain, that foster unhealthy behaviors (e.g., poor diet, physical inactivity, underage drinking) and hinder weight management. A “spillover effect” may also operate, whereby even “true never‐smokers”, those who would not have initiated smoking regardless, may benefit from healthier peer environments (C. Courtemanche 2009).

Consistent with prior evidence of reduced initiation, we estimate that T21 laws increase the probability of being a “never smoker” by 4.4 percentage points. Conditioning on smoking status in repeated cross‐sectional data such as the BRFSS raises potential selection concerns, because the composition of “never smokers” may differ before and after T21 adoption. However, our main results using the full BRFSS sample, which is randomly drawn and not compositionally affected by T21,^21^ remain statistically significant. Because “never smokers” comprise 83.7% of the full sample and no significant effects are found among “ever smokers”,^22^ we infer that the estimated weight management effects are largely driven by “never smokers”.

### 5.2 YRBS Findings

**FIGURE 3:**
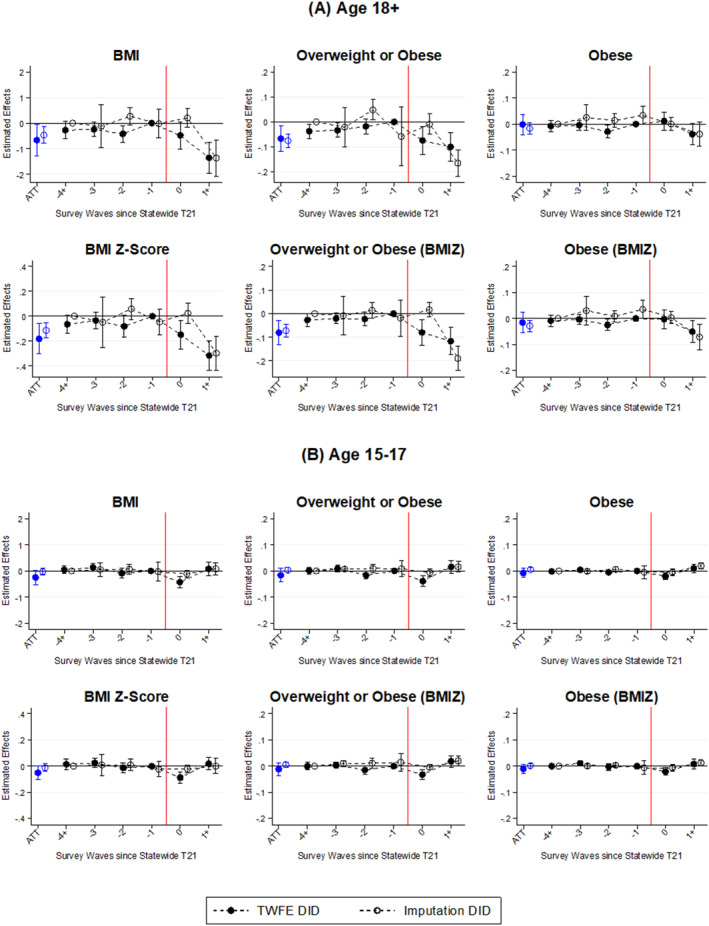
Effects of T21 laws on body weight among high schoolers, YRBS, 2009–2019. ATT: average treatment effects on the treated; BMI: body mass index; BMIZ: body mass index Z‐score; DID: difference‐in‐differences; TWFE: two‐way fixed‐effects.

Figure 3 presents ATT and event study estimates of T21's effects on body weight using YRBS data. Panel (A) reports results for high schoolers aged 18+. TWFE ATT estimates (solid blue circles) show significant reductions in BMI, BMI Z‐scores (standardized BMI percentiles), and the probability of being overweight or obese under both BMI and Z‐score definitions. Corresponding TWFE event study estimates (solid black circles) indicate that these declines emerge in the first post‐T21 survey wave and strengthen thereafter. A significant reduction in obesity is observed after the first post‐T21 survey wave. For comparison, hollow circles report imputation DID estimates, which are broadly consistent with the TWFE results, except that first‐wave effects are no longer statistically significant while ATT estimates for obesity become significant.

Overall, these results suggest that T21's weight management effects among high schoolers aged 18+ are more pronounced near the overweight cutoff, interestingly aligning with the BRFSS results for young adults without HSD. Mirroring this pattern, quantile regressions in Table 2, Panel B, show significant effects at the 0.5, 0.6, and 0.7 quantiles, corresponding to centile levels of 23.0–25.6. Quantile regressions on BMI Z‐scores yield similar patterns (not reported for brevity).

Supporting Information S1: Table A4 presents subsample analyses by gender and race for YRBS sample, showing effects concentrated among male students, consistent with our BRFSS results and Hansen et al. (2023). While effects on all body weight outcomes are more pronounced among non‐Hispanic White students, non‐White students also experience a statistically significant decline in the probability of being overweight or obese.

We also examine whether T21's effects on body weight extend to adolescents aged 15–17. As shown in Figure 3, Panel (B), TWFE ATT estimates indicate statistically significant but much smaller reductions in BMI and BMI Z‐scores. Event study estimates show small declines in all body weight outcomes in the first post‐T21 survey wave that effects fade thereafter. The Imputation DID estimates are even smaller and close to zero. Table 2, Panel C, shows generally weaker effects across BMI quantiles (and BMI Z‐score quantiles, not reported for brevity) than for students aged 18+, but with any detectable effects larger at higher quantiles.

### 5.3 Weight‐Related Behaviors

We examine behavioral mechanisms underlying T21's weight management effects using multiple data sources. Table 4, Panel A, presents results for our BRFSS sample. Replicating Hansen et al. (2023), we find T21 laws significantly reduce smoking participation and daily smoking by 3.6 percentage points (−32.5%) and 2.7 percentage points (−44.2%), respectively. We also find T21 laws lead to a small (but barely significant, *p* = 0.104) increase in exercise participation, a higher probability of meeting USDA daily fruit intake guidelines, a decline in binge drinking, and improved mental health as indicated by reduced FMD. As another placebo test, we also examine weight‐related outcomes unlikely to be directly affected by T21 laws, such as health insurance coverage and medical care affordability, and find no significant effects.

**TABLE 4:** Effects of T21 laws on weight‐related behaviors, 2009–2019, DID estimates.

Panel A: BRFSS 18−20	Current smoking	Everyday smoking	Exercise	Fruit	Vegetable	Current drinking	Binge drinking	FMD	Health insurance	Medical care
	(1)	(2)	(3)	(4)	(5)	(6)	(7)	(8)	(9)	(10)
	−0.036***	−0.027***	0.021	0.045**	0.014	−0.036	−0.023*	−0.017*	0.020	−0.001
	(0.008)	(0.006)	(0.013)	(0.020)	(0.009)	(0.025)	(0.014)	(0.009)	(0.018)	(0.012)
	[−32.5%]	[−44.2%]	[2.4%]	[4.6%]	[1.4%]	[−10.0%]	[−14.1%]	[−14.3%]	[2.4%]	[−1.0%]

**Panel B:** **YRBS** **18+**	**Current smoking**	**Frequent smoking**	**Exercise**	**Fruit**	**Vegetable**	**Current drinking**	**Excessive TV**	**Heavy Soda**	**Marijuana use**	**Frequent marijuana**
	**(1)**	**(2)**	**(3)**	**(4)**	**(5)**	**(6)**	**(7)**	**(8)**	**(10)**	**(10)**
	−0.008	−0.056***	0.039	0.007	−0.041	0.008	−0.076***	−0.038**	−0.036*	−0.066***
	(0.033)	(0.017)	(0.043)	(0.021)	(0.052)	(0.030)	(0.028)	(0.017)	(0.019)	(0.017)
	[−4.3%]	[−86.5%]	[4.8%]	[2.2%]	[−4.0%]	[1.7%]	[−26.9%]	[−73.2%]	[−13.6%]	[−52.8%]

*Note:* All regressions use survey‐provided sampling weights. Robust standard errors in parenthesis are clustered at the state level. Percent changes relative to corresponding pre‐treatment means for treated states are reported in brackets. Regressions in Panel A control respondents' age (along with age‐specific time trends), gender, race/ethnicity, educational attainment, student status, and employment status, as well as logarithmic group average income. Regressions in Panel B control binary indicators for respondents' gender, race/ethnicity, and grades. All regressions control cigarette taxes, state population coverages of comprehensive ISR by venue (bars, restaurants, and workplaces), state population coverages of comprehensive IVR by venue (bars, restaurants, and workplaces), standardized e‐cigarette taxes, presence of MLPA for e‐cigarettes at 18, beer taxes, medical marijuana laws, recreational marijuana laws, gasoline taxes, soda sales taxes, food sales taxes, state PE time requirements, state nutrition education standards, state school beverage nutrition standards by venue (cafeterias, vending machines, school stores, fundraisers), personal income per capita, and unemployment rates. Regressions in Panel A control state fixed effects and year‐by‐month fixed effects. Regressions in Panel B control state fixed effects and year fixed effects.

^*^ *p* < 0.1.

^**^ *p* < 0.05.

^***^ *p* < 0.01.

**FIGURE 4:**
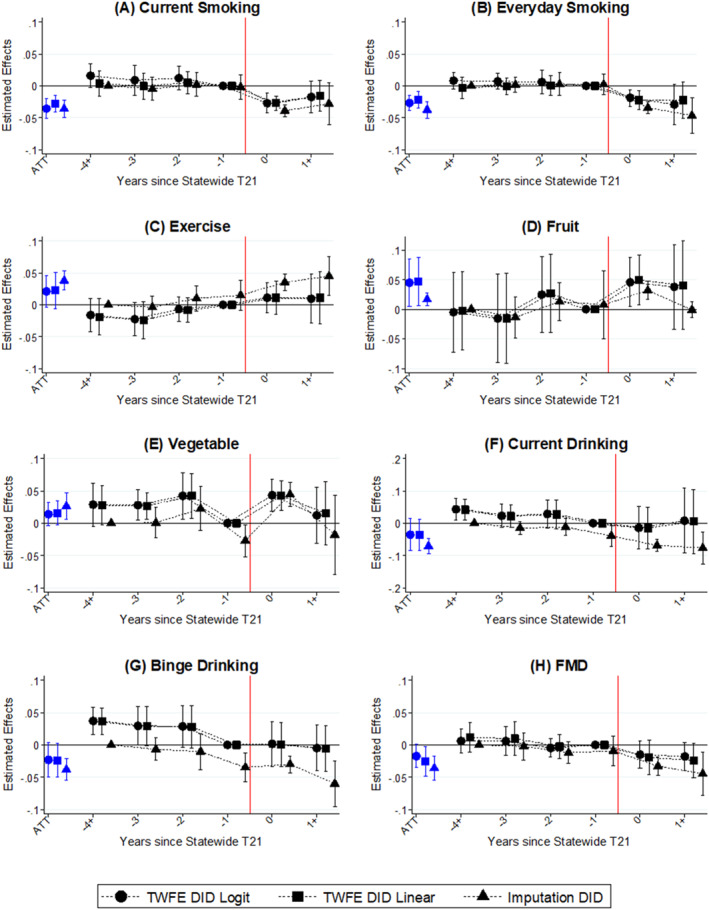
Effects of T21 laws on weighted‐related behaviors among young adults aged 18–20, BRFSS, 2009‐2019. ATT: average treatment effects on the treated; DID: difference‐in‐differences; FMD: frequent mental distress; TWFE: two‐way fixed‐effects.

To unpack the dynamics of these behavioral responses, Figure 4 plots event study estimates (alongside ATT estimates). Circles denote TWFE Logit marginal effects, our preferred specification for behavioral outcomes as in Table 4. Squares and triangles are TWFE LPM and imputation DID estimates, respectively, for comparison. Logit and LPM estimates are very similar across outcomes. All specifications show consistent declines in current and daily smoking following T21 adoption. While post‐treatment effects on exercise participation are statistically insignificant in the TWFE specifications, the imputation DID estimates indicate a significant increase. All specifications show increases in fruit and vegetable intake in the first post‐T21 year. Interestingly, the imputation DID estimates reveal significant post‐T21 reductions in alcohol drinking, but with anticipation effects that may explain the attenuation of TWFE estimates.^23^ Reductions in FMD are consistently observed across specifications.

Table 4, Panel B, presents mechanism results for high schoolers aged 18+ in YRBS. Again, consistent with Hansen et al. (2023), T21 laws significantly reduce frequent smoking but not overall smoking participation. While effects on exercise, fruit and vegetable intake, and alcohol drinking are statistically insignificant, we find significant declines in excessive TV watching and heavy soda consumption. We also replicate Hansen et al. (2023)'s finding that T21 laws reduce marijuana use, which may contribute to improved weight outcomes given biomedical evidence linking marijuana use to increased appetite and caloric intake (Greenberg et al. 1976; Mattes et al. 1994; Sansone and Sansone 2014). Meanwhile, evidence shows that marijuana legalization is associated with lower body weight (Sabia et al. 2017), implying a likewise complex relationship between marijuana use, marijuana regulations, and body weight. Nonetheless, the observed declines in unhealthy behaviors suggest that T21 laws may help prevent harmful habits. Event study estimates for YRBS behavioral outcomes are presented in Supporting Information S1: Figure A5. Results are generally consistent across Logit and LPM specifications,^24^ but only the Logit model reproduces the significant post‐T21 decline in frequent smoking as in Hansen et al. (2023).

Subsample results for BRFSS and YRBS weight‐related behaviors are reported in Supporting Information S1: Table A5. Behavioral improvements are generally more pronounced in subgroups exhibiting stronger weight management effects, including young adults with BMI≥25, consistent with the main findings on body weight.

We additionally examine weight‐related daily activities using 2009‐2019 ATUS data. Because some outcomes like daily exercise minutes have many zeros, we estimate a two‐part model, using Logit for participation and negative binomial for conditional duration. Supporting Information S1: Appendix B provides details on our ATUS sample (Supporting Information S1: Table A6) and methods. Supporting Information S1: Table A7 reports coefficient estimates from each part in Columns (1)–(2) and overall marginal effects in Column (3), and Supporting Information S1: Figure A6 presents event study estimates of the two‐part model marginal effects. We find significant increases in exercise time and reductions in sedentary leisure time. Time spent eating, particularly eating at home, increases significantly in the first post‐T21 year and then attenuates, while the conditional duration of eating out declines in the same period. Spending more time eating, alongside a shift toward at‐home meals from dining out, may support healthier weight outcomes (Restrepo and Zeballos 2020).

### 5.4 Exploratory Evidence From the Post‐Federal T21 Period

Our main analysis follows the literature in focusing on pre‐federal T21 period through 2019 (Hansen et al. 2023; Abouk et al. 2024; C. Cotti et al. 2024; Bersak et al. 2025). After the federal T21 law took effect in December 2019, its enforcement varied across states, and many states continued to adopt their own statewide T21 laws (Abouk et al. 2024). As of 2025, only seven states remain without a statewide T21 law, motivating an exploratory analysis of post‐federal effects of statewide T21 laws.

Identifying post‐federal statewide T21 effects is empirically challenging because states without statewide laws often claim compliance with the federal law, leaving few clean control states. Nevertheless, we extend our BRFSS and YRBS data through 2023 and still define treatment by statewide T21 adoption, implicitly assuming weak federal enforcement. We additionally control for flavored tobacco sales restrictions (Donovan et al. 2025) and COVID‐19 incidence.

Supporting Information S1: Figure A7 reports results for BRFSS body weight and smoking outcomes. Using the full 2009‐2023 sample (left panels), obesity reductions remain similar to our main findings, with additional declines in “overweight or obese” and average BMI, but effects on smoking disappear. Estimates from 2020–2023 (middle panels) are highly noisy, even after excluding pre‐federal adopters. Allowing longer pre‐treatment periods for the remaining post‐federal adopters (right panels) yields more stable estimates but shows no significant effects on smoking or BMI, aside from a transitory decline in “overweight or obese”. Supporting Information S1: Figure A8 reports results for the YRBS. Estimates based on the 2021–2023 waves (middle panels) are extremely noisy and implausibly large. With longer pre‐treatment periods (right panels), smoking outcomes show significant reductions, whereas body weight effects are all statistically insignificant. We note that these results are exploratory and should be interpreted with caution.

In addition, extending data into the post‐federal period allows us to re‐estimate the stacked DID models while retaining all 17 pre‐federal adopters (excluding post‐federal adopters). The extended stacked DID estimates shown in Supporting Information S1: Figure A9 continue to qualitatively support our main findings of reduced adverse weight outcomes.

## 6 Discussion

A central motivation of our study is to assess whether T21 laws, a prevention‐oriented tobacco regulation, lead to adverse weight gain often associated with cessation. Our results indicate they do not. We find no evidence that T21 laws worsen body weight outcomes among young adults. Meanwhile, we do not find broad weight reductions either. Instead, we find that statewide T21 adoption leads to a 2.1‐percentage‐point decline in the probability of being obese, a 17.9% decline relative to the pre‐treatment obesity prevalence for this age group in treated states. These results reflect that T21‐induced weight reductions are concentrated in the upper tail of the BMI distribution, a pattern later made explicit by quantile regression results. The implied BMI reductions at upper quantiles are modest by themselves (e.g., a 1.1‐unit, 3.6% decrease at the 0.9 quantile), but they occur near the obesity cutoff and therefore translate into a noticeable decline in obesity in our BRFSS sample.^25^

Our main findings are robust across extensive robustness checks and falsification tests, including modern DID estimators (e.g., imputation DID) that address staggered adoption. Exploring heterogeneity further, we find that the effects are stronger among males and non‐White young adults. We detect no heterogeneity by income. Individuals with and without HSD both experience reductions in adverse weight outcomes but concentrated in different upper segments of the BMI distribution. The effects are driven primarily by “never smokers”, aligning with a prevention‐based pathway. Supplemental YRBS analysis finds similar reductions in adverse weight outcomes among high school students aged 18+, but weight reductions among adolescents aged 15–17 are weaker and transitory.

Our behavioral evidence points to plausible mechanisms. In BRFSS, T21 laws are associated with increased exercise participation and fruit/vegetable intake, alongside reductions in alcohol drinking and mental distress. In YRBS, T21 laws reduce excessive TV watching, heavy soda consumption, and marijuana use. ATUS evidence shows increased exercise time, less sedentary leisure, and a shift toward at‐home eating from dining‐out. Together, these findings suggest that T21 laws encourage healthier lifestyles and discourage downstream risky behaviors beyond smoking.

We acknowledge some limitations of this study. First, as noted earlier, subgroup analyses conditional on smoking status using repeated cross‐sectional data may raise selection concerns. A natural alternative is to use longitudinal PATH data like in C. Cotti et al. (2024). However, public‐use PATH reports adult age only in broad intervals (e.g., 18–24). Future work applying restricted‐use PATH can address this limitation. This would also allow researchers to study how T21 affects tobacco use intensity among continuing users and related body weight effects. Nonetheless, large sample, repeated cross‐sectional data like the BRFSS are still widely acceptable for evaluating “population‐level” T21 effects. Second, our post‐federal period exploratory analysis implicitly assumes weak federal T21 enforcement and in fact lacks clean control states. Future research should examine heterogeneity in federal enforcement and disentangle federal versus statewide T21 effects.

Despite these limitations, this study contributes to the tobacco‐obesity literature by providing the first causal evidence that T21 laws, a prevention‐oriented tobacco control policy, do not generate the adverse weight gain often associated with cessation and instead improve weight outcomes among young adults at higher risk of obesity. By preventing initiation among youth and young adults, T21 laws steer them away from clusters of unhealthy behaviors that often come with smoking and create better peer environments that may benefit those unlikely to smoke regardless (C. Courtemanche 2009).

More broadly, this study offers important insights for economists and policymakers beyond tobacco issues. Our findings suggest that early‐life, prevention‐oriented substance control policies can achieve goals without the unintended side effects often associated with corrective interventions, while also improving related behaviors and generating larger welfare gains. This speaks to broader economic discussions on behavioral complementarities and the welfare implications of public policies. From a policy perspective, such youth‐focused prevention policies may be cost‐effective complements to conventional interventions. Their multi‐dimensional public health benefits can be further enhanced when paired with other health initiatives (e.g., fitness, nutrition, mental health services) and when tailored to heterogeneous behavioral responses across subpopulations.

## Funding

The authors have nothing to report.

## Conflicts of Interest

The authors declare no conflicts of interest.

## Supporting information


Supporting Information S1

## Data Availability

The data that support the findings of this study are available from the corresponding author upon reasonable request.

## Endnotes

^1^
Body Mass Index = Weight(kg)/Height(m)^2^.

^2^
DeCicca et al. (2022) provide a comprehensive review of the impacts of tobacco regulations.

^3^
Before T21, most states set the MLPA for tobacco at 18. Alabama, Alaska, and Utah set it at 19. Hansen et al. (2023) detail the history of tobacco MLPA and T21 laws in the U.S., which we briefly summarize in Section 2.1.

^4^
The National Academy of Medicine estimates that T21 laws could prevent 223,000 deaths among cohorts born between 2000 and 2019.

^5^
Supporting Information S1: Table A1 lists statewide T21 enactment dates and each state's population coverage of local T21 laws prior to statewide or federal adoption. Over 500 localities enacted T21 laws before the higher‐level implementation.

^6^
International studies have similar findings (Fang et al. 2009; Sen et al. 2010; Amialchuk et al. 2018).

^7^
USDA dietary guidelines recommend that females (males) in this age group consume 1.5 (2) cups of fruits and 2 (3) cups of vegetables daily (Lee et al. 2022).

^8^
We construct smoking indicators following Hansen et al. (2023). As in their study, only 16.3% of our sample report “having ever smoked 100+ cigarettes in lifetime”, meaning that 83.7% are classified as “never smokers.”

^9^
We impute average income grouped by race and education in each state‐year to mitigate potential reverse causality with body weight (Ruhm 2005).

^10^
YRBS defines exercise as ≥ 60 min of activity, unlike BRFSS, which counts any physical activity.

^11^
We hold local T21 population coverage at its maximum pre‐statewide level after statewide adoption (see Supporting Information S1: Table A1), ranging from 0% (no local T21) in some states to 74.5% in New York. Some states without statewide T21 laws like Alaska and Missouri exceeded 40% local T21 coverage by the time of federal adoption.

^12^
Supporting Information S1: Appendix A describes the sources and measures/definitions of all state‐level covariates used in this paper.

^13^
Results for binary body weight outcomes are robust to alternative Logit models (Supporting Information S1: Figure A1).

^14^
When outcome probabilities are close to 0, LPM can perform poorly, and nonlinear models are preferable (Von Hippel 2015; Greene and Wilson 2021).

^15^
Recent work on staggered adoption DID cautions against relying solely on pre‐trend estimates to assess parallel trends (De Chaisemartin and d’Haultfoeuille, 2020; Goodman‐Bacon 2021; Callaway and Sant’Anna 2021; Sun and Abraham 2021; Baker et al. 2022). In Sections 5.1.2, we show that our results are robust to modern DID approaches that address staggered adoption, especially the imputation DID estimator of Borusyak et al. (2024).

^16^
We exclude state‐specific trends from the event study in Figure 1A due to multicollinearity concerns. Including them raises the variance inflation factor (VIF) to 19.7 (compared to 8.9 without), exceeding the commonly cited threshold of 10 that warns multicollinearity (Wooldridge 2012, 98). While point estimates remain qualitatively similar when state‐specific trends are included (Supporting Information S1: Figure A2), standard errors unsurprisingly increase. In the static DID specification for ATT estimates, adding state‐specific trends raises the VIF from 4.4 to 5.7.

^17^
In Section 5.4, we show that extending the study period into the post‐federal T21 era allows us to retain all 17 pre‐federal adopting states, yielding more precise stacked DID estimates that remain consistent with our main findings.

^18^
It applies an event study design with a continuous treatment (Callaway et al. 2024).

^19^
Small positive point estimates appear at lower quantiles, but they are statistically insignificant and lie within the normal weight range. In additional analyses (available upon request), we find that T21 laws have no significant effect on overweight status among individuals with BMI < 30 but reduce the probability of obesity by 6.6 percentage points (20%) among those with BMI ≥ 25, consistent with our main findings and the quantile regression results.

^20^
The effect is especially stronger among non‐Hispanic blacks and Hispanics (available upon request).

^21^
Supporting Information S1: Table A3 reports BRFSS covariate balance tests, showing no significant differences in most demographics. After applying a Bonferroni correction for multiple hypothesis testing, no differences remain statistically significant, indicating no meaningful changes in sample composition around T21 implementation.

^22^
We also observe a statistically significant obesity reduction among “former smokers”. However, given their small sample size (*N* = 3800; 4% of the full sample), it's unlikely that this group drives the main results.

^23^
Such anticipation effects may reflect retailer training and stricter ID‐checking practices prior to formal T21 enforcement (Muralidharan et al. 2019), which could plausibly spill over to underage drinking control.

^24^
Since YRBS is biennial and not all states participate in every wave, state‐year cells are sparse. Moreover, some behavioral outcomes become very rare in later survey waves (e.g., frequent smoking near 2% in 2019). These features undermine the reliability of linear‐based imputation DID estimators, which depend on precisely estimated cell means and are sensitive to sampling noise when cells are sparse and outcomes are rare. Thus, we do not report imputation DID results for YRBS behavioral outcomes.

^25^
Our estimates are no larger than those in prior literature. C. Courtemanche (2009) finds that a $1‐per‐pack increase in cigarette prices/taxes reduces obesity by up to 3.6 percentage points, whereas Baum (2009) implies a similarly sized obesity increase. To benchmark magnitudes, we provide a back‐of‐the‐envelope translation of T21 adoption into a cigarette‐price/tax‐equivalent change based on smoking reduction. T21 lowers youth smoking by 32.5%. Assuming a price elasticity of −0.7 and a $2.49/pack average pre‐treatment price (C. Courtemanche 2009), this implies a 46% (32.5%/0.7) or $1.15/pack (0.46 × 2.49) price increase, making our estimates comparable to the $1‐cigarette‐price/tax effects. Other binary‐form tobacco regulations, such as workplace bans and randomized cessation treatment, have been shown to generate similar or larger weight responses, albeit in the opposite direction (Liu et al. 2010; C. Courtemanche et al. 2018). While relative changes in obesity may appear larger considering lower youth obesity prevalence, we note that prior studies often focus on single tobacco products (e.g., cigarettes), whereas T21 targets all tobacco use and may have broader impacts.
