# Supplementary material for: Beyond Tobacco Prevention: The Effects of Tobacco 21 Laws on Young Adults' Body Weight
Source: Health Econ. 2026 Jun 22;35(9):1406–23. doi: 10.1002/hec.70120 (PMC13428626; doi:10.1002/hec.70120)
Supplement: Supplementary file 1 — Supporting Information S1 [file HEC-35-1406-s001.docx]

**Appendix A: Data Sources and Measures/Definitions of State-level Control Variables**

| **Variables** | **Measures / Definitions** | **Sources and links (if available)** |
| --- | --- | --- |
| State population coverage of local T21 laws | % | University of Michigan T21 Population Coverage Database  <https://sph.umich.edu/cseph/helper/our-research/t21-data.html> |
| ***Other Tobacco Control Policies*** | | |
| Cigarette taxes | $/pack | Tax Policy Center  <https://taxpolicycenter.org/statistics/state-cigarette-tax-rates> |
| State population coverage of comprehensive ISR by venue (bars, restaurants, and workplaces - separately) | % | Seidenberg, A. B., Braganza, K., Chomas, M., Diaz, M. C., Friedman, A. S., Phillips, S., & Pesko, M. (2024). Coverage of Indoor Smoking and Vaping Restrictions in the US, 1990–2021. *American Journal of Preventive Medicine*, *67*(4), 494-502. |
| State population coverage of comprehensive IVR by venue (bars, restaurants, and workplaces - separately) | % |  |
| Presence of indoor smoking bans on K-12 campus | binary | State Tobacco Activities Tracking and Evaluation (STATE) System Legislation (custom report) |
| Presence of indoor smoking bans on university campus | binary |  |
| E-cigarette taxes | $/ml | Cotti, C., Nesson, E., Pesko, M. F., & Phillips, S. (2024). Standardising the measurement of e-cigarette tax rates in the USA , 2010–2023. *Tobacco Control*. |
| Presence of MLPA for e-cigarettes at 18 | Binary | State Tobacco Activities Tracking and Evaluation (STATE) System Legislation (custom report) |
| ***Alcohol and Marijuana Policies*** | | |
| Beer excise taxes | $/gallon | Tax Policy Center  <https://www.taxpolicycenter.org/statistics/state-alcohol-excise-tax-rates> |
| Presence of medical marijuana laws | binary | Insurance Institute of Highway Safety  <https://www.iihs.org/research-areas/alcohol-and-drugs/marijuana-laws-table> |
| Presence of recreational marijuana laws | binary |  |
| ***Weight-related Policies*** | | |
| Gasoline taxes | $/gallon | Tax Policy Center  <https://www.taxpolicycenter.org/statistics/state-motor-fuels-tax-rates> |
| Soda sales taxes | % | Bridging the Gap <https://bridgingthegap.ihrp.uic.edu/research/sodasnack_taxes/index.html> [Note: this link has expired, but we had the data.]  Supplemented by Tax Foundation  <https://taxfoundation.org/data/all/state/sales-tax-rates/>  <https://taxfoundation.org/data/all/state/halloween-candy-tax-groceries-soda-sales-tax/> |
| Food sales taxes | % |  |

(Continue)

| **Variables** | **Measures / Definitions** | **Sources and links (if available)** |
| --- | --- | --- |
| ***Weight-related Policies*** | | |
| High school Physical education (PE) time requirement | Binary ($\geq$60min/wk) | National Cancer Institute  <https://class.cancer.gov/StateProfiles> |
| High school Nutrition education standards | Binary (Curriculum required) |  |
| High school beverage nutrition standards by venue (cafeterias, vending machines, school stores, fundraisers - separately) | Binary  (Meet/exceed federal guidelines) |  |
| ***Economic Conditions*** | | |
| State personal income per capita | $ | Bureau of Economic Analysis  <https://www.bea.gov/itable/regional-gdp-and-personal-income> |
| State unemployment rates | % | Bureau of Labor Statistics Local Area Unemployment Statistics <https://data.bls.gov/cgi-bin/dsrv?la> |
| ***Additional Controls for Extended Study Periods to 2023*** | | |
| State population coverage of comprehensive flavored tobacco sales restrictions (by product) | % | Donovan, E. M., Braganza, K., Diaz, M. C., Seidenberg, A. B., Kreslake, J. M., & Pesko, M. F. (2025). Population coverage and comprehensiveness of flavoured tobacco sales restrictions in the USA, 2010–2023. *Tobacco Control*. |
| State COVID-19 incidence | cases per 100,000 population | CDC COVID-19 Data Tracker  <http://covid.cdc.gov/covid-data-tracker>  U.S. Census Bureau population estimates  <https://www.census.gov/topics/population/data.html> |

**Appendix B: Description of ATUS Data and Methods**

As another supplemental analysis of weight-related behaviors, we use data from the 2009-2019 American Time Use Survey (ATUS), a nationally representative cross-sectional survey on how Americans allocate their daily time. We focus on young adults aged 18-20. Given the relatively small sample size (N=4,170), the ATUS results should be interpreted as suggestive.

Table A6 presents summary statistics for the ATUS sample. On average, young adults report 15 minutes of daily exercise, though 86% report no exercise at all (not shown). To account for exercise intensity, we convert exercise minutes into metabolic equivalent of task (MET) values,^[[1]](#footnote-1)^ which average 72 minutes daily. Respondents also spend 262 minutes (over 4 hours) per day in sedentary leisure (excluding sleep) and 85 minutes eating (excluding food preparation), including 65 minutes eating at home and 20 minutes eating out.

Due to the high prevalence of zeros for variables like daily exercise minutes, we estimate a two-part model based on Equation (1), consisting of a Logit model for participation and a negative binomial model for conditional duration. Due to the small sample size, we estimate at annual level to avoid sparse cells, but we control month fixed effects and day-of-week fixed effects. We also estimate corresponding event study estimates for marginal effects from the two-part model.

**Appendix C: Ancillary Tables and Figures**

**Table A1. Statewide T21 Laws Effective Dates and Local T21 Population Coverage before Statewide or Federal T21 Laws**

| **States** | **Statewide**  **T21 Laws**  **Effective Dates** | **Local T21 Population Coverage before Statewide T21 Laws** | **Local T21 Population Coverage before Federal T21 Law** |
| --- | --- | --- | --- |
| ***17 states that adopted statewide T21 laws before federal T21*** | | | |
| Hawaii | 1-01-2016 | 13.8% |  |
| California | 6-09-2016 | 0.2% |  |
| District of Columbia | 2-18-2017 |  |  |
| New Jersey | 11-01-2017 | 9.0% |  |
| Oregon | 1-01-2018 | 9.1% |  |
| Maine | 7-01-2018 | 5.0% |  |
| Massachusetts | 12-31-2018 | 72.0% |  |
| Illinois | 7-01-2019 | 37.8% |  |
| Virginia | 7-01-2019 |  |  |
| Delaware | 7-16-2019 |  |  |
| Arkansas | 9-01-2019 | 1.0% |  |
| Texas | 9-01-2019 | 5.4% |  |
| Vermont | 9-01-2019 |  |  |
| Connecticut | 10-01-2019 | 17.2% |  |
| Maryland | 10-01-2019 |  |  |
| Ohio | 10-16-2019 | 17.1% |  |
| New York | 11-13-2019 | 74.6% |  |
| ***27 states that adopted statewide T21 laws after federal T21*** | | | |
| Washington | 1-1-2020 |  |  |
| Kentucky | 3-26-2020 |  |  |
| Oklahoma | 5-19-2020 |  |  |
| Iowa | 6-29-2020 |  |  |
| Wyoming | 7-1-2020 |  |  |
| Pennsylvania | 7-1-2020 |  |  |
| South Dakota | 7-1-2020 |  |  |
| Utah | 7-1-2020 | 2.7% | 2.5% |
| Indiana | 7-1-2020 |  |  |
| Mississippi | 7-8-2020 |  |  |
| Colorado | 7-14-2020 | 22.5% | 17.2% |
| Georgia | 7-22-2020 | 0.5% |  |
| New Hampshire | 7-30-2020 | 6.5% | 6.5% |
| Minnesota | 8-1-2020 | 51.3% | 34.7% |

(Continue Table A1)

| **States** | **Statewide**  **T21 Laws**  **Effective Dates** | **Local T21 Population Coverage before Statewide T21 Laws** | **Local T21 Population Coverage before Federal T21 Law** |
| --- | --- | --- | --- |
| Nebraska | 8-15-2020 |  |  |
| New Mexico | 1-1-2021 |  |  |
| Tennessee | 1-1-2021 |  |  |
| Nevada | 5-27-2021 |  |  |
| Rhode Island | 7-7-2021 | 3.6% | 3.4% |
| North Dakota | 8-1-2021 |  |  |
| Louisiana | 8-1-2021 |  |  |
| Alabama | 8-1-2021 |  |  |
| Florida | 10-1-2021 | 3.6% | 2.1% |
| Idaho | 7-1-2022 |  |  |
| Michigan | 7-21-2022 |  |  |
| Kansas | 7-1-2023 | 34.0% | 32.5% |
| West Virginia | 6-7-2024 |  |  |
| ***7 states without statewide T21 as of 2025*** | | | |
| Alaska |  |  | 41.1% |
| Arizona |  |  | 2.6% |
| Missouri |  |  | 41.8% |
| Montana |  |  |  |
| North Carolina |  |  |  |
| South Carolina |  |  |  |
| Wisconsin |  |  |  |

*Note*: Statewide Tobacco 21 (T21) law effective dates are from Tobacco21.org. Data on local T21 population coverage are from the University of Michigan T21 Population Coverage Database. For states without statewide T21 adoption during our study period, empty cells indicate 0% local coverage.

**Table A2. Summary Statistics for BRFSS and YRBS Samples**

|  | **Weighted Mean (St. D.)** | |
| --- | --- | --- |
|  | **BRFSS 18-20** | **YRBS 18+** |
|  | **[N=88,534]** | **[N=86,472]** |
| ***Outcome Variables*** |  |  |
| BMI | 24.37 (4.73) | 24.50 (5.37) |
| Overweight or Obese | 0.357 (0.479) | 0.358 (0.479) |
| Obese | 0.124 (0.330) | 0.137 (0.344) |
| Exercise participation | 0.852 (0.355) | 0.811 (0.392) |
| Adequate fruit intake (meet USDA standard) | 0.294 (0.456) | 0.295 (0.456) |
| Adequate vegetable intake (meet USDA standard) | 0.210 (0.407) | 0.193 (0.395) |
| Excessive TV watching (3+ hours per school day) |  | 0.272 (0.445) |
| Heavy soda intake (>3 times per day) |  | 0.056 (0.231) |
| Current smoking | 0.118 (0.322) | 0.178 (0.383) |
| Frequent smoking |  | 0.065 (0.247) |
| Everyday smoking | 0.069 (0.253) |  |
| Current drinking | 0.336 (0.472) | 0.429 (0.495) |
| Binge drinking | 0.155 (0.362) |  |
| Frequent mental distress | 0.134 (0.341) |  |
| Marijuana use |  | 0.258 (0.437) |
| Frequent marijuana use |  | 0.120 (0.325) |
|  |  |  |
| ***Demographic Covariates*** |  |  |
| Age 18 | 0.373 (0.484) |  |
| Age 19 | 0.318 (0.466) |  |
| Age 20 | 0.309 (0.462) |  |
| Female | 0.464 (0.499) | 0.460 (0.498) |
| White, non-Hispanic | 0.547 (0.498) | 0.514 (0.500) |
| Black, non-Hispanic | 0.128 (0.335) | 0.179 (0.383) |
| Hispanic | 0.211 (0.408) | 0.231 (0.422) |
| Other races, non-Hispanic | 0.113 (0.317) | 0.076 (0.265) |
| Some high school | 0.166 (0.372) |  |
| High school graduate | 0.466 (0.499) |  |
| Some college | 0.360 (0.480) |  |
| Student | 0.490 (0.500) |  |
| Employed | 0.373 (0.484) |  |
| Group average household income ($) | 49,184 (16,878) |  |
| Grade 11 |  | 0.081 (0.272) |
| Grade 12 |  | 0.907 (0.291) |

*Note:* Summary statistics use data-provided sampling weights.

**Table A3. BRFSS Covariate Balance Tests**

| **Variables** | **Estimated Effects (St. Errors)** |
| --- | --- |
| Age 19 | 0.016 (0.016) |
| Age 20 | 0.004 (0.012) |
| Female | -0.012 (0.009) |
| Black, non-Hispanic | 0.017 (0.017) |
| Hispanic | -0.025 (0.011)^**^ |
| Other races, non-Hispanic | 0.003 (0.015) |
| High school graduate | 0.001 (0.012) |
| Some high school | -0.004 (0.008) |
| Student | -0.016 (0.009)^*^ |
| Employed | 0.002 (0.011) |
| *Ln* (group average household income) | -0.008 (0.017) |

*Note*: The balance test is conducted by regressing each covariate on the statewide T21 indicator, controlling other individual demographics, state covariates, state fixed effects, and year-month fixed effects. Regressions include individuals in 50 states and the District of Columbia (N=88,534). All regressions use BRFSS-provided sampling weights. Robust standard errors in parenthesis are clustered at the state level. State covariates include cigarette taxes, state population coverages of comprehensive ISR by venue (bars, restaurants, and workplaces), state population coverages of comprehensive IVR by venue (bars, restaurants, and workplaces), standardized e-cigarette taxes, presence of MLPA for e-cigarettes at 18, beer taxes, medical marijuana laws, recreational marijuana laws, gasoline taxes, soda sales taxes, food sales taxes, state PE time requirements, state nutrition education standards, state school beverage nutrition standards by venue (cafeterias, vending machines, school stores, fundraisers), personal income per capita, and unemployment rates. *** *p*<0.01, ** *p*<0.05, * *p*<0.1.

**Table A4. Effects of T21 Laws on Body Weight among High School Students Aged 18+, YRBS, 2009-2019, Subsample by Gender and Race, DID Estimates**

|  | **BMI** | **Overweight or Obese** | **Obese** | **BMI**  **Z-Scores** | **Overweight or Obese (BMIZ)** | **Obese**  **(BMIZ)** |  |
| --- | --- | --- | --- | --- | --- | --- | --- |
|  | **(1)** | **(2)** | **(3)** | **(4)** | **(5)** | **(6)** | **# Obs.** |
| ***By Gender*** | | | | | | | |
| Male | -1.150^***^  (0.399)  [-4.6%] | -0.141^***^  (0.036)  [-34.9%] | -0.024  (0.026)  [-16.8%] | -0.331^***^  (0.095)  [-26.8%] | -0.178^***^  (0.035)  [-54.5%] | -0.050^*^  (0.027)  [-29.6%] | 47,202 |
| Female | -0.198  (0.406)  [-0.8%] | 0.008  (0.029)  [2.5%] | 0.023  (0.025)  [18.7%] | -0.025  (0.076)  [-2.4%] | 0.025  (0.035)  [9.0%] | 0.023  (0.023)  [21.5%] | 39,270 |
| ***By Race/Ethnicity*** | | | | | | | |
| White | -0.980^***^  (0.344)  [-4.1%] | -0.131^***^  (0.043)  [-41.8%] | 0.009  (0.021)  [8.5%] | -0.310^***^  (0.077)  [-27.8%] | -0.131^***^  (0.039)  [-52.7%] | -0.037  (0.031)  [-32.4%] | 49,307 |
| Nonwhite | -0.792  (0.572)  [-3.2%] | -0.070^*^  (0.037)  [-16.8%] | -0.010  (0.036)  [-6.3%] | -0.208^*^  (0.107)  [-18.1%] | -0.078^**^  (0.037)  [-22.5%] | -0.010  (0.038)  [-6.8%] | 37,165 |

*Note*: Regressions include YRBS sampled high school students aged 18+ in 44 states (N=86,472). All regressions use YRBS -provided sampling weights. Robust standard errors in parenthesis are clustered at the state level. Regressions control binary indicators for respondents’ gender, race/ethnicity, and grades, local T21 population coverage, cigarette taxes, state population coverages of comprehensive ISR by venue (bars, restaurants, and workplaces), state population coverages of comprehensive IVR by venue (bars, restaurants, and workplaces), standardized e-cigarette taxes, presence of MLPA for e-cigarettes at 18, beer taxes, medical marijuana laws, recreational marijuana laws, gasoline taxes, soda sales taxes, food sales taxes, state PE time requirements, state nutrition education standards, state school beverage nutrition standards by venue (cafeterias, vending machines, school stores, fundraisers), personal income per capita, unemployment rates, state fixed effects, and year fixed effects. *** *p*<0.01, ** *p*<0.05, * *p*<0.1.

**Table A5. Effects of T21 Laws on Weight-Related Behaviors, 2009-2019, Subsample Analysis, TWFE Logit Marginal Effects**

| **Panel A: BRFSS 18-20** | **Current**  **Smoking** | **Everyday Smoking** | **Exercise** | **Fruit** | **Vegetable** | **Current Drinking** | **Binge Drinking** | **FMD** |
| --- | --- | --- | --- | --- | --- | --- | --- | --- |
|  | **(1)** | **(2)** | **(3)** | **(4)** | **(5)** | **(6)** | **(7)** | **(8)** |
| ***By Gender*** |  |  |  |  |  |  |  |  |
| Male | -0.037^***^  (0.012) | -0.032^***^  (0.008) | 0.013  (0.017) | 0.041  (0.028) | 0.026  (0.019) | -0.017  (0.021) | -0.032  (0.021) | -0.016^**^  (0.008) |
| Female | -0.035^***^  (0.011) | -0.020^***^  (0.007) | 0.029  (0.021) | 0.043  (0.038) | -0.038^**^  (0.016) | -0.054  (0.045) | -0.014  (0.022) | -0.021  (0.017) |
| ***By Race*** |  |  |  |  |  |  |  |  |
| White | -0.065^***^  (0.022) | -0.047^***^  (0.011) | -0.003  (0.011) | 0.008  (0.036) | -0.009  (0.028) | -0.032  (0.022) | -0.019  (0.020) | -0.048^***^  (0.017) |
| Nonwhite | -0.011  (0.007) | -0.010^*^  (0.006) | 0.048^*^  (0.026) | 0.100^***^  (0.029) | 0.012  (0.020) | -0.027  (0.044) | -0.033^*^  (0.017) | 0.014  (0.022) |
| ***By Income*** |  |  |  |  |  |  |  |  |
| High Income | -0.078  (0.050) | -0.042  (0.029) | -0.047  (0.031) | 0.289^***^  (0.098) | 0.071^*^  (0.039) | -0.068  (0.063) | 0.026  (0.051) | -0.024  (0.015) |
| Low Income | -0.025^***^  (0.009) | -0.020^***^  (0.006) | 0.036^*^  (0.020) | 0.027  (0.025) | -0.006  (0.017) | -0.016  (0.026) | -0.022^*^  (0.012) | -0.009  (0.010) |
| ***By Education*** |  |  |  |  |  |  |  |  |
| With HSD | -0.023^**^  (0.009) | -0.015^**^  (0.007) | 0.023  (0.017) | 0.023  (0.020) | 0.014  (0.019) | -0.024  (0.026) | -0.015  (0.013) | -0.017^**^  (0.008) |
| Without HSD | -0.132^***^  (0.028) | -0.115^***^  (0.021) | 0.019  (0.053) | 0.236^**^  (0.096) | -0.060  (0.042) | -0.120^**^  (0.053) | -0.081^**^  (0.036) | -0.016  (0.031) |
| ***By BMI*** |  |  |  |  |  |  |  |  |
| BMI$\geq$25 | -0.023^**^  (0.011) | -0.031^***^  (0.010) | 0.030  (0.020) | -0.055^**^  (0.022) | 0.049^*^  (0.027) | -0.084^**^  (0.040) | -0.041^***^  (0.015) | -0.008  (0.019) |
| BMI<25 | -0.039^***^  (0.013) | -0.026^***^  (0.007) | 0.002  (0.011) | 0.089^**^  (0.037) | -0.013  (0.024) | -0.007  (0.020) | -0.003  (0.021) | -0.013  (0.011) |
| **Panel B:**  **YRBS 18+** | **Current Smoking** | **Frequent Smoking** | **Exercise** | **Excessive TV** | **Heavy**  **Soda** | **Current Drinking** | **Marijuana Use** | **Frequent**  **Marijuana** |
|  | **(1)** | **(2)** | **(3)** | **(4)** | **(5)** | **(6)** | **(7)** | **(8)** |
| ***By Gender*** |  |  |  |  |  |  |  |  |
| Male | -0.055  (0.046) | -0.099^***^  (0.026) | -0.010  (0.035) | -0.114^***^  (0.030) | -0.074^***^  (0.026) | -0.032  (0.039) | -0.062^**^  (0.030) | -0.107^***^  (0.026) |
| Female | 0.039  (0.029) | -0.018  (0.022) | 0.084  (0.052) | -0.053  (0.039) | 0.016  (0.016) | 0.042  (0.035) | -0.008  (0.028) | -0.024  (0.018) |
| ***By Race*** |  |  |  |  |  |  |  |  |
| White | -0.055  (0.037) | -0.052  (0.040) | -0.074^***^  (0.023) | -0.077  (0.063) | -0.185^***^  (0.047) | -0.016  (0.036) | 0.010  (0.027) | -0.052^***^  (0.019) |
| Nonwhite | 0.009  (0.045) | -0.039^***^  (0.012) | 0.160^**^  (0.063) | 0.003  (0.034) | -0.034  (0.026) | 0.007  (0.032) | -0.113^***^  (0.038) | -0.097^***^  (0.020) |

*Note*: All regressions use survey-provided sampling weights. Robust standard errors in parenthesis are clustered at the state level. Regressions in Panel A control respondents’ age (along with age-specific time trends), gender, race/ethnicity, educational attainment, student status, and employment status, as well as logarithmic group average income. Regressions in Panel B control binary indicators for respondents’ gender, race/ethnicity, and grades. All regressions control cigarette taxes, state population coverages of comprehensive ISR by venue (bars, restaurants, and workplaces), state population coverages of comprehensive IVR by venue (bars, restaurants, and workplaces), standardized e-cigarette taxes, presence of MLPA for e-cigarettes at 18, beer taxes, medical marijuana laws, recreational marijuana laws, gasoline taxes, soda sales taxes, food sales taxes, state PE time requirements, state nutrition education standards, state school beverage nutrition standards by venue (cafeterias, vending machines, school stores, fundraisers), personal income per capita, and unemployment rates. Regressions in Panel A control state fixed effects and year-by-month fixed effects. Regressions in Panel B control state fixed effects and year fixed effects. *** *p*<0.01, ** *p*<0.05, * *p*<0.1.

**Table A6. Summary Statistics for ATUS Samples**

|  | **Weighted Mean (Standard Deviation)** |
| --- | --- |
|  | **[N=4,170]** |
| ***Dependent Variables*** |  |
| Minutes doing exercise per day | 14.9 (64.7) |
| MET value of minutes doing exercise per day | 71.8 (317.0) |
| Minutes relaxing sedentarily per day | 262.1 (303.0) |
| Minutes eating per day | 84.8 (123.5) |
| Minutes eating at home per day | 65.1 (98.9) |
| Minutes eating out per day | 19.7 (82.0) |
| ***Demographic Characteristics*** |  |
| Female | 0.484 (0.500) |
| White, non-Hispanic | 0.559 (0.497) |
| Black, non-Hispanic | 0.108 (0.311) |
| Hispanic | 0.260 (0.439) |
| Other races, non-Hispanic | 0.073 (0.259) |
| Some high school | 0.338 (0.473) |
| High school graduate | 0.321 (0.467) |
| Some college | 0.336 (0.472) |
| Group average household income ($) | 84,743 (60,728) |
| Employed | 0.534 (0.499) |
| Student | 0.600 (0.490) |

*Note:* Summary statistics use ATUS-provided sampling weights.

**Table A7. Effects of T21 Laws on Weight-Related Daily Activities among Young Adults Aged 18-20, ATUS, 2009-2019, Two-Part Model**

|  | **Estimated**  **Coefficients** | | **Marginal**  **Effects** |
| --- | --- | --- | --- |
| **Dependent**  **Variables** | **First Part**  **(Logit)** | **Second Part**  **(Negative Binomial)** |  |
|  | **(1)** | **(2)** | **(3)** |
| MET value of minutes doing exercise | -0.169  (0.803) | 0.880^***^  (0.218) | 38.53  (34.87)  [82.2%] |
| Minutes relaxing sedentarily | -0.143  (0.280) | -0.187  (0.119) | -50.61^*^  (45.41)  [-20.1%] |
| Minutes eating | 1.428^**^  (0.610) | 0.378^**^  (0.163) | 47.19^***^  (15.06)  [58.5%] |
| Minutes eating at home | 1.106^***^  (0.354) | 0.322^*^  (0.169) | 36.44^***^  (11.83)  [56.1%] |
| Minutes eating out | 0.112  (0.364) | -0.591^**^  (0.278) | -10.03  (7.40)  [-63.6%] |

*Note*: All regressions use ATUS-provided sampling weights. Robust standard errors in parenthesis are clustered at the state level. Percent changes relative to pre-treatment means for treated states are reported in brackets. Regressions control respondents’ age (along with age-specific time trends), gender, race/ethnicity, educational attainment, student status, employment status, logarithmic group average income, cigarette taxes, state population coverages of comprehensive ISR by venue (bars, restaurants, and workplaces), state population coverages of comprehensive IVR by venue (bars, restaurants, and workplaces), standardized e-cigarette taxes, presence of MLPA for e-cigarettes at 18, beer taxes, medical marijuana laws, recreational marijuana laws, gasoline taxes, soda sales taxes, food sales taxes, state PE time requirements, state nutrition education standards, state school beverage nutrition standards by venue (cafeterias, vending machines, school stores, fundraisers), personal income per capita, unemployment rates, state fixed effects, year fixed effects, month fixed effects, and day-of-week fixed effects. *** *p*<0.01, ** *p*<0.05, * *p*<0.1.

**
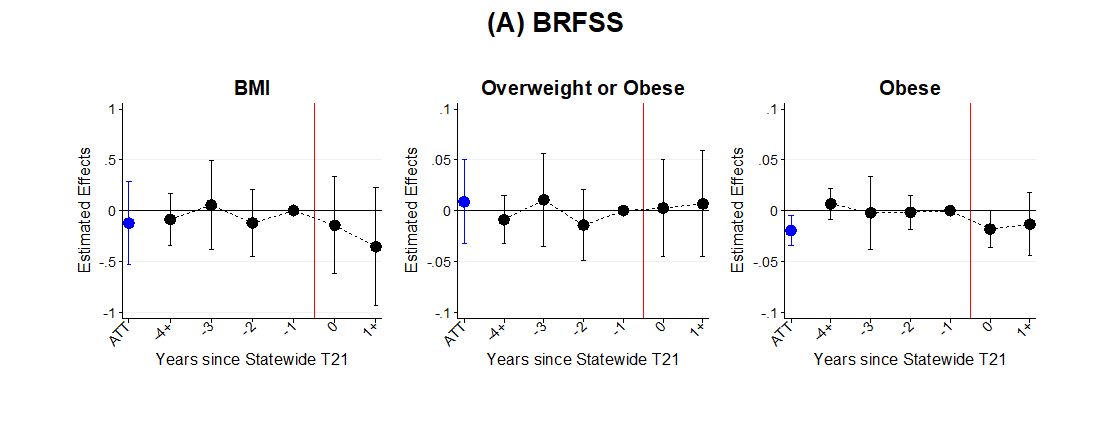
**

**
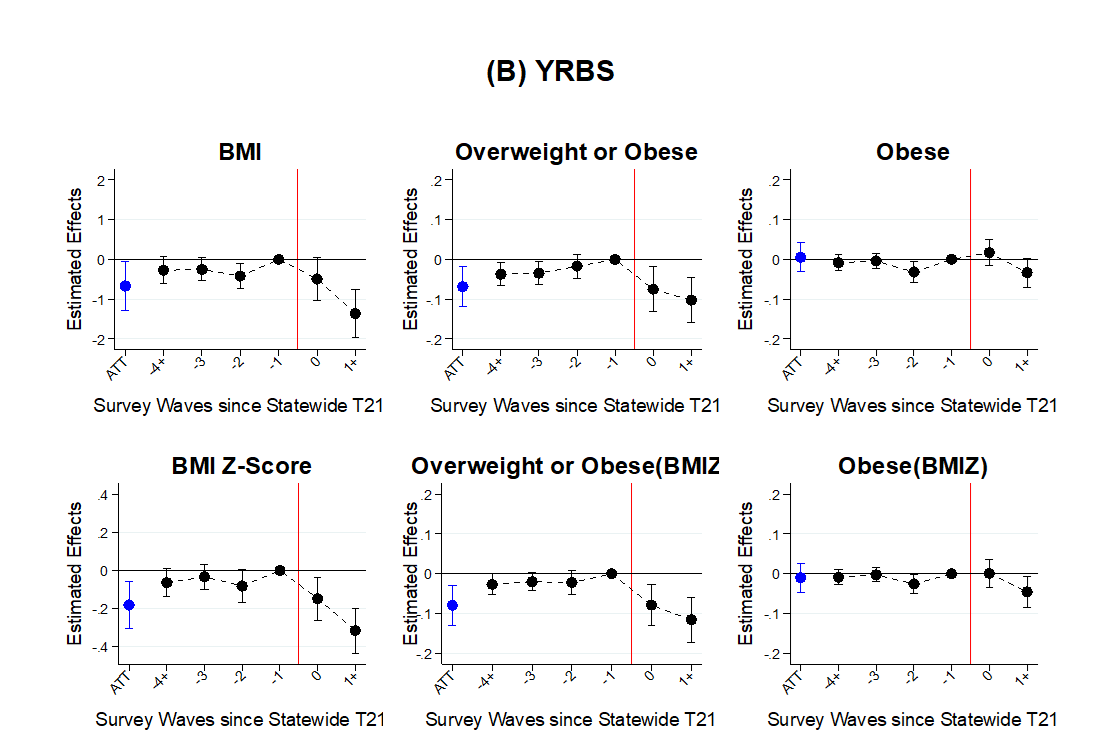
**

**Figure A1. Effects of T21 Laws on Body Weight, 2009-2019, Logit Models for Binary Outcomes**

**
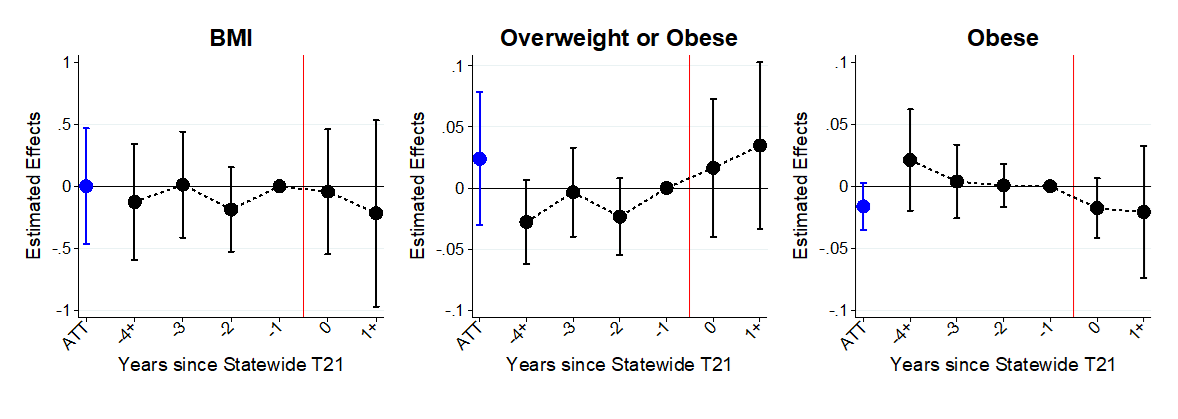
**

**Figure A2. Effects of T21 Laws on Body Weight among Young Adults Aged 18-20, BRFSS, 2009-2019, State-Specific Time Trends**

**
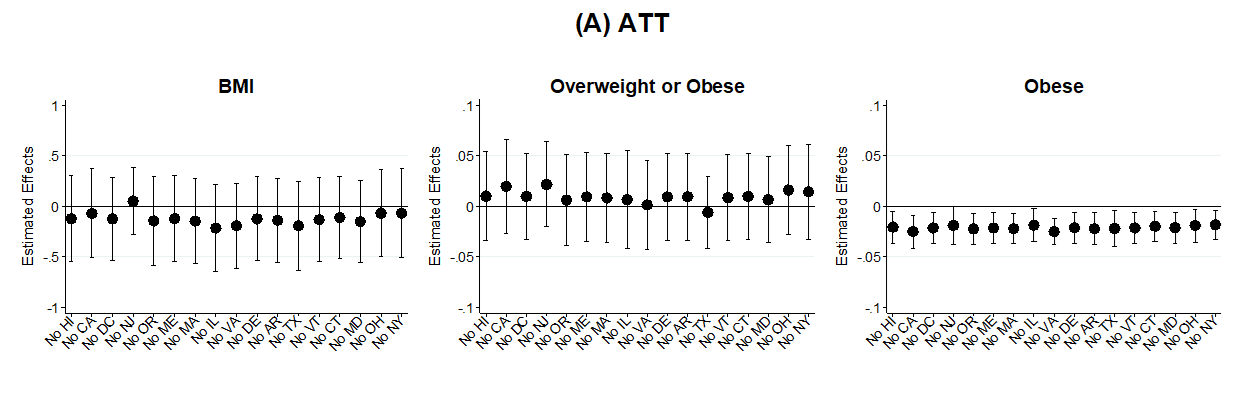
**

**
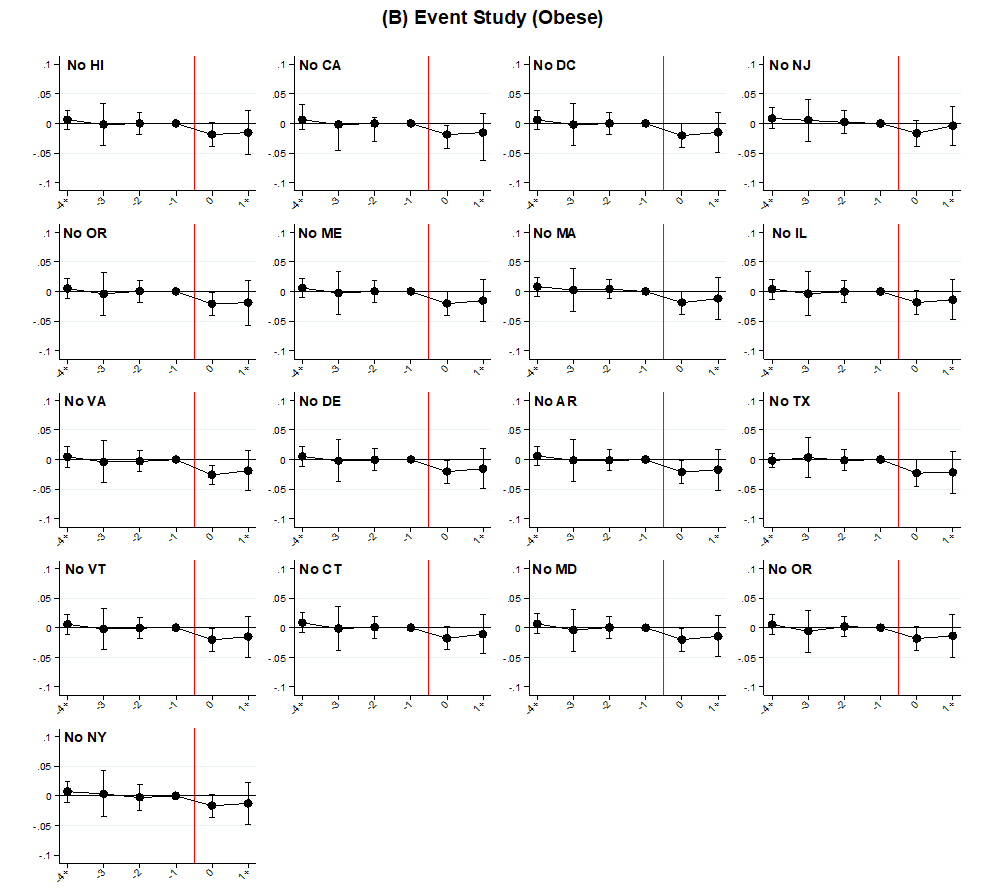
**

**Figure A3. Effects of T21 Laws on Body Weight among Young Adults Aged 18-20, BRFSS, 2009-2019, Leave-One-Treated-State-Out Robustness Check**

**
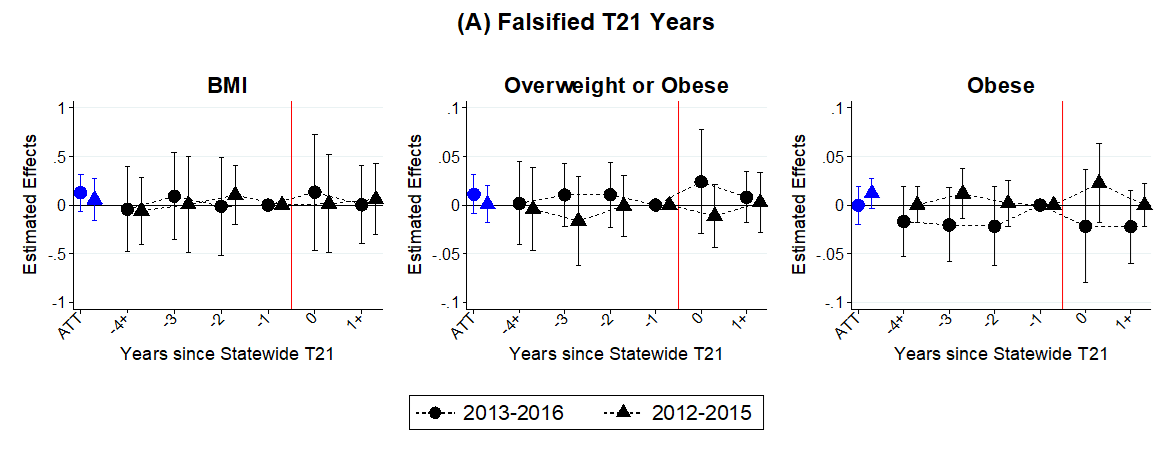
**

**
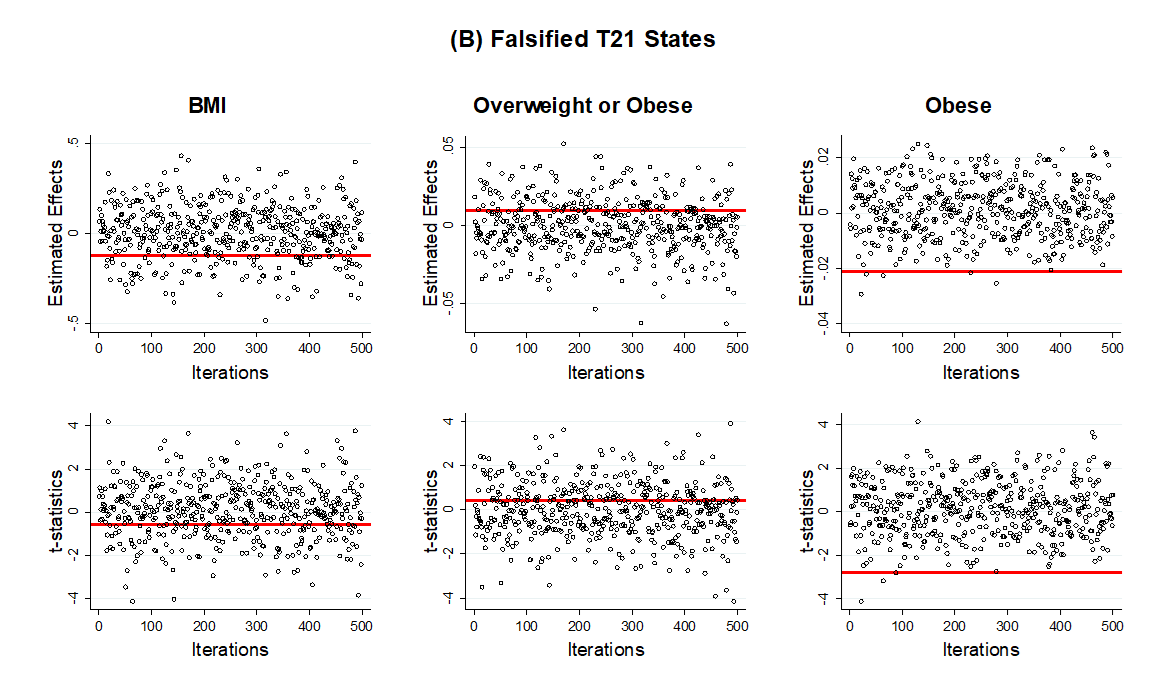
 Figure A4. Effects of T21 Laws on Body Weight among Young Adults Aged 18-20, BRFSS, 2009-2019, Falsified Timing and Falsified States**

**
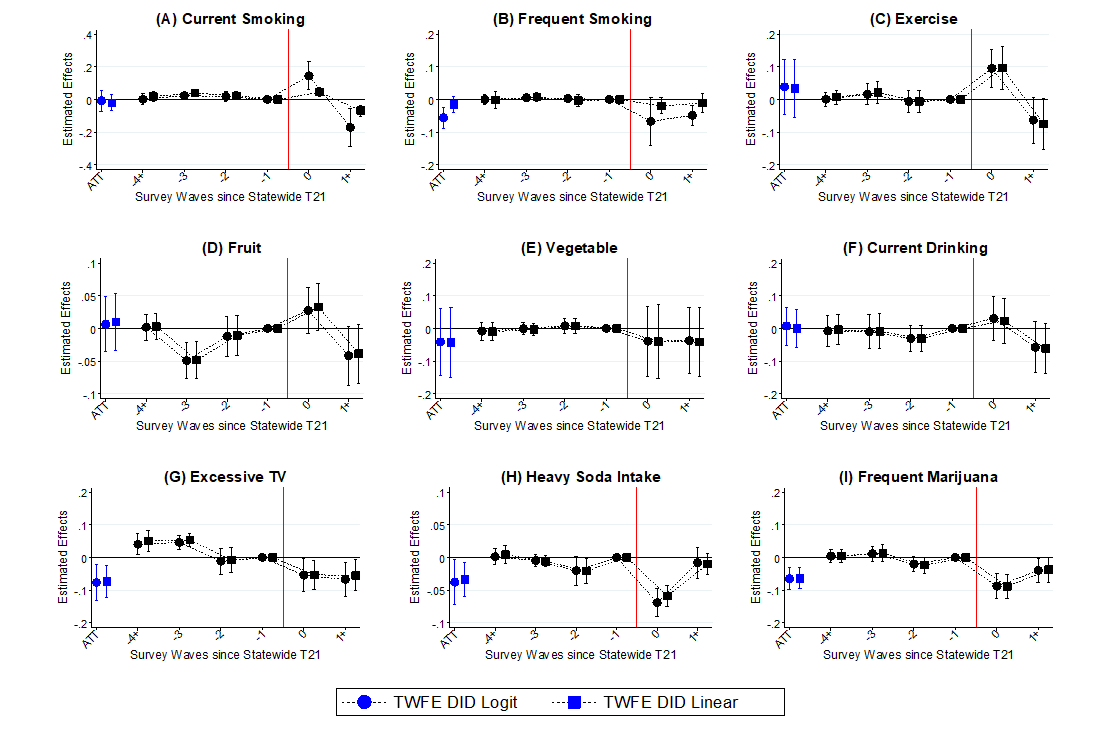
**

**Figure A5. Effects of T21 Laws on Weight-Related Behaviors among High Schoolers Aged 18+, YRBS, 2009-2019**

**
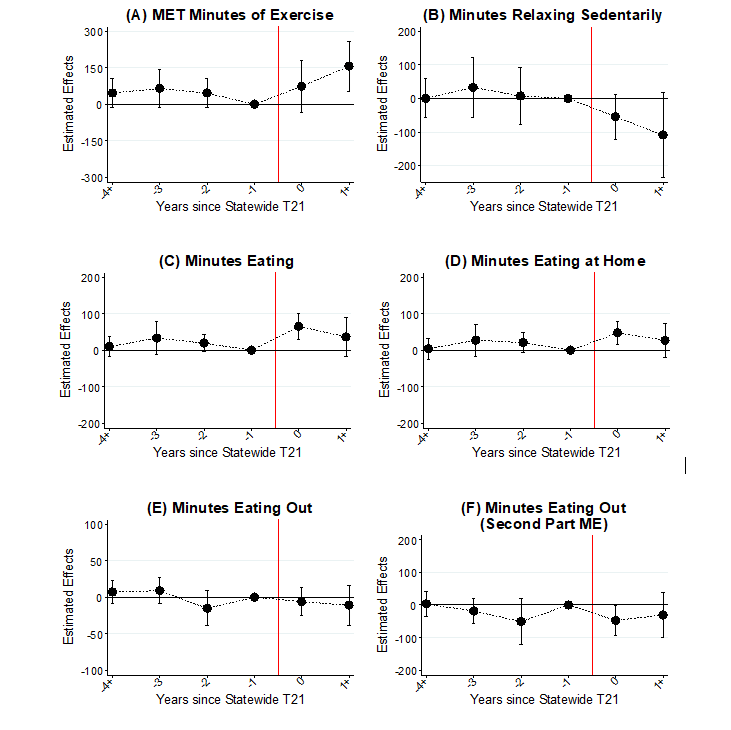
**

**Figure A6. Effects of T21 Laws on Weight-Related Daily Activities among Young Adults Aged 18-20, ATUS, 2009-2019, Two-Part Model Marginal Effects**


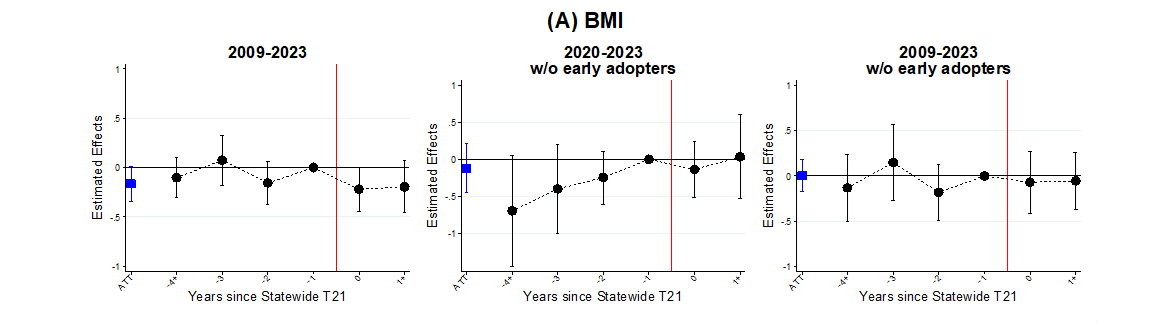

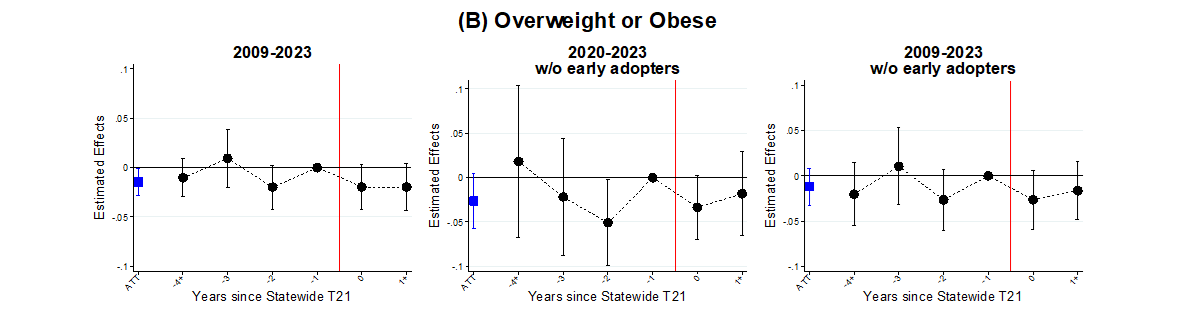

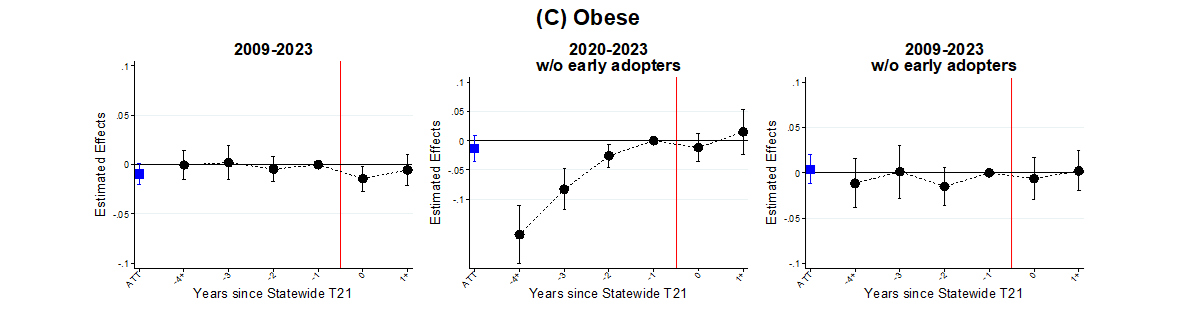

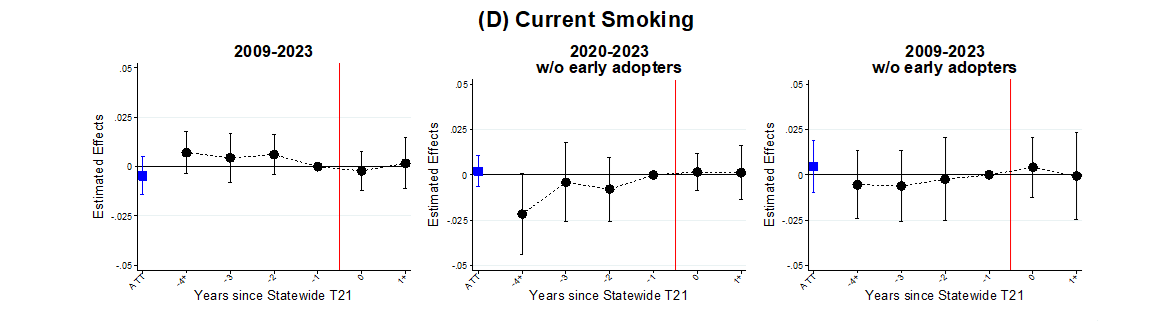

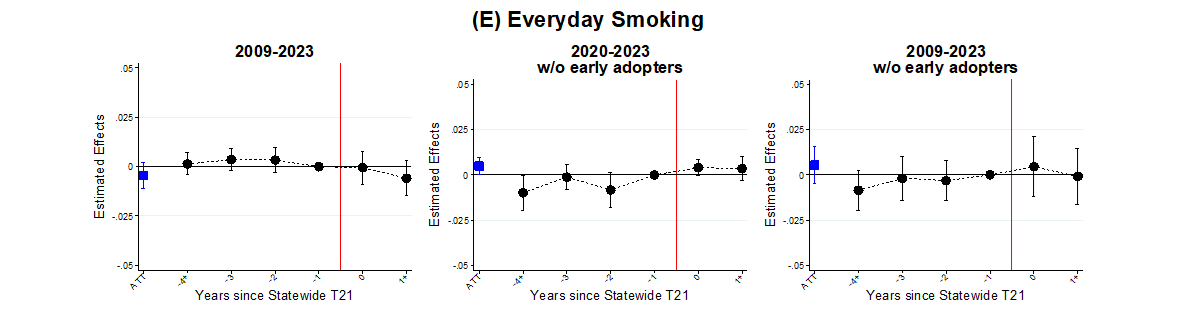


**Figure A7. Effects of T21 Laws on Body Weight and Smoking among Young Adults Aged 18-20, BRFSS, 2009-2023**


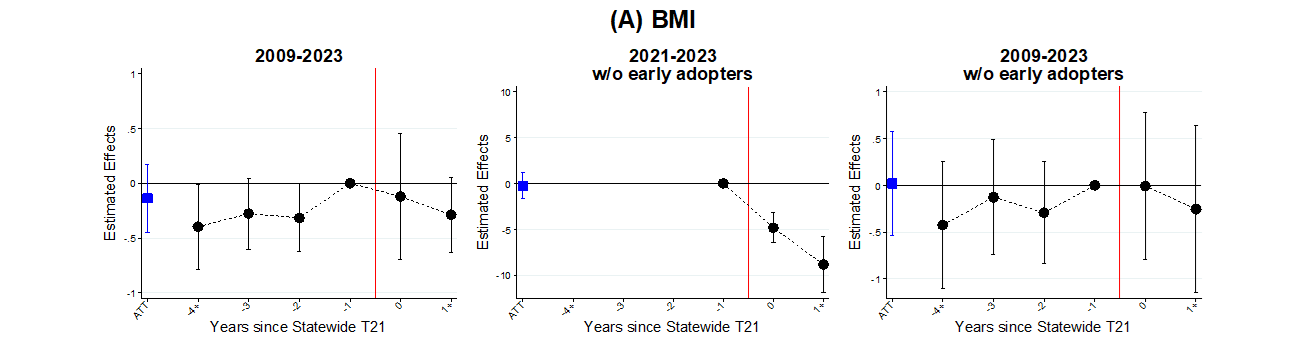

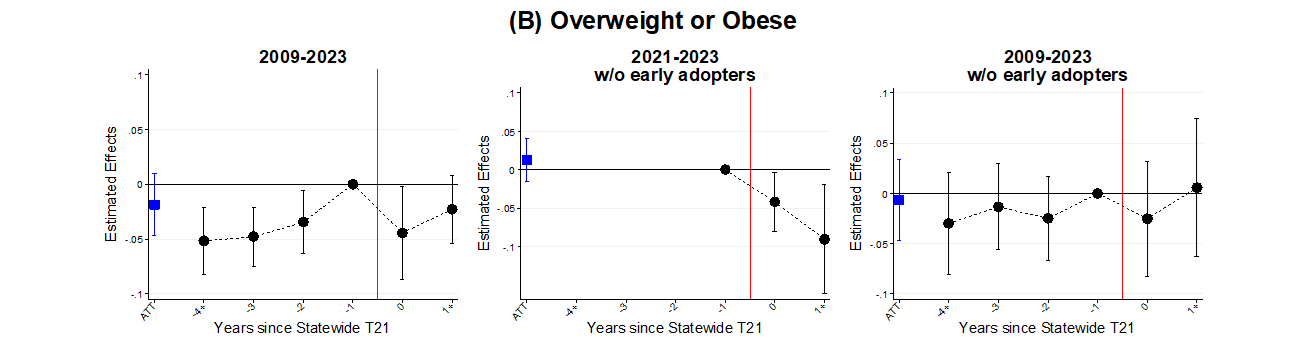

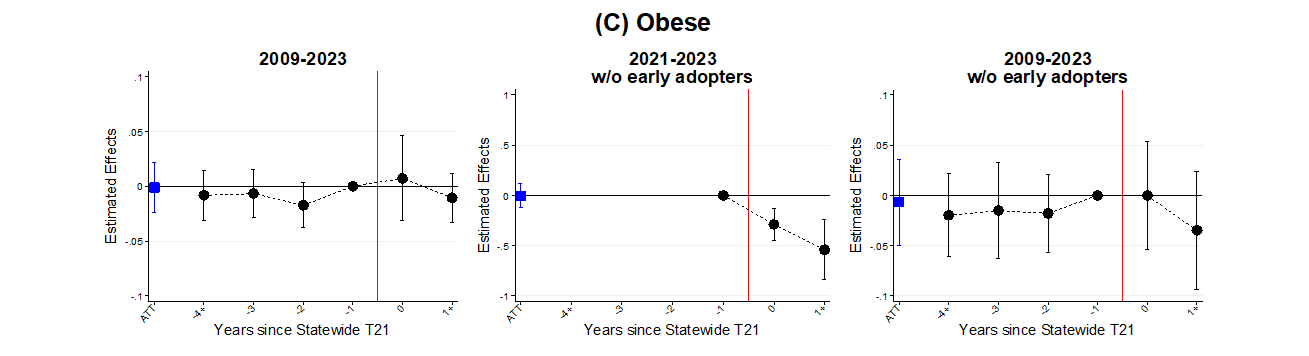

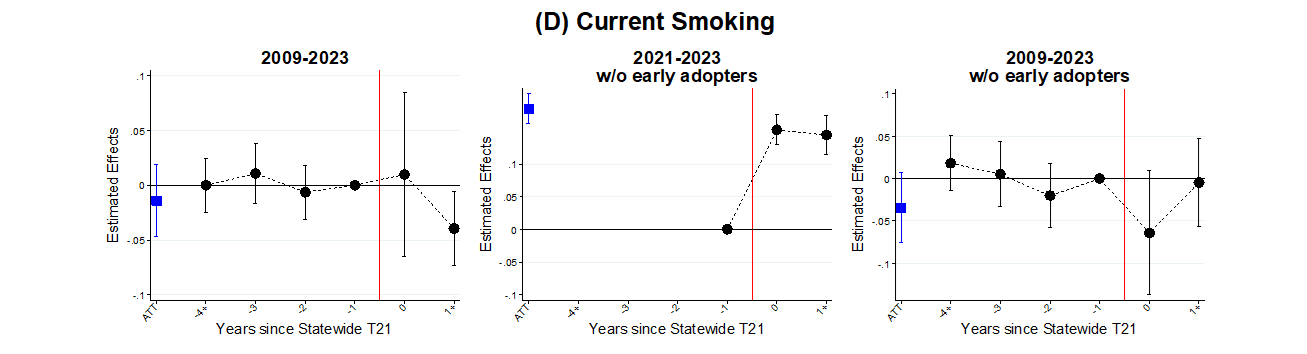

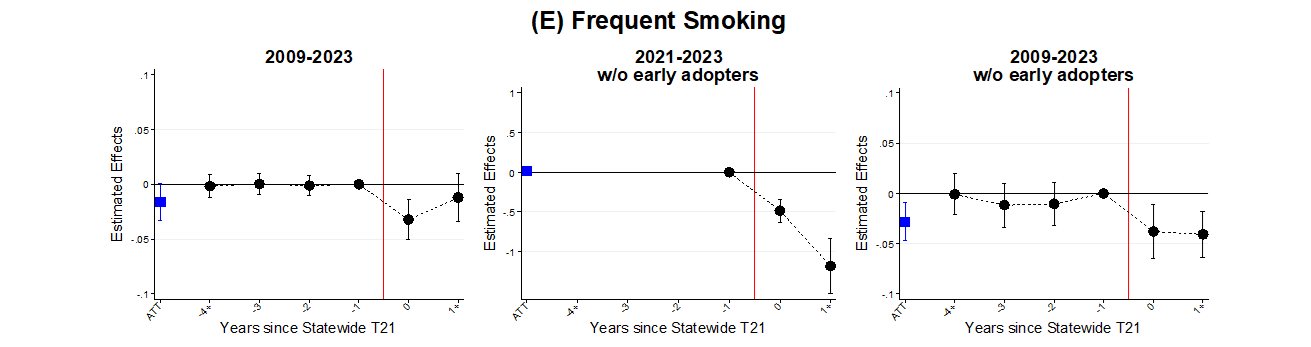


**Figure A8. Effects of T21 Laws on Body Weight and Smoking among High Schoolers Aged 18+, YRBS, 2009-2023**

**
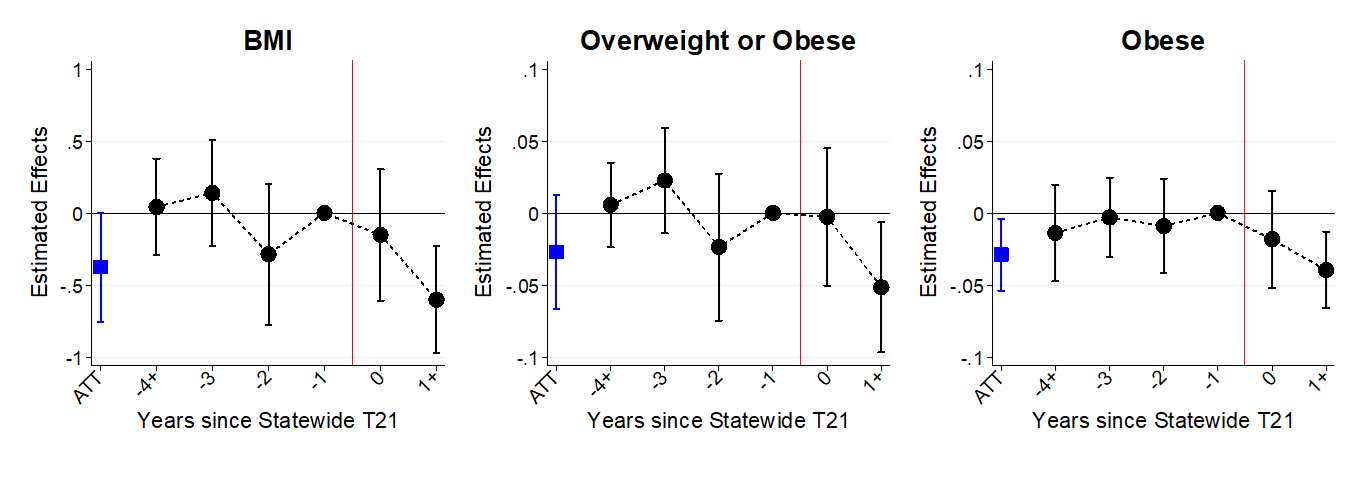
**

**Figure A9. Effects of T21 Laws on Body Weight among Young Adults Aged 18-20, BRFSS, Stacked DID with All 17 Pre-Federal Adopters**

1. MET is defined as working metabolic rate relative to resting metabolic rate (Dave and Yang, 2022). [↑](#footnote-ref-1)
